# Modeling process instability in anaerobic digestion by using a thermodynamically corrected model

**DOI:** 10.3389/fbioe.2026.1880446

**Published:** 2026-08-04

**Authors:** Rebecca Wittum, Arne Naegel, Gabriel Wittum

**Affiliations:** 1 TechSim UG, Ölbronn-Dürrn, Germany; 2 MSQC, Goethe University of Frankfurt, Frankfurt(Main), Germany; 3 Computer, Electrical and Mathematical Sciences and Engineering, KAUST, Thuwal, Saudi Arabia

**Keywords:** anaerobic digestion, Gibbs energy, inhibition processes, modeling, process instability, thermodynamic

## Abstract

Mathematical modeling of anaerobic digestion requires consideration of many different aspects. One of these is the computation of biochemical reaction rates under varying conditions, where probing mechanisms for process instabilities are of special importance in finding optimal efficiency. This work implements a model with thermodynamic considerations applied to all aceto- and methanogenic processes on the basis of respective Gibbs free energies. It demonstrates their necessity to reflect the accumulation of longer-chain acids in some cases of process instability. For this purpose, the results of different computational setups are compared to experimental data for methane production and acid concentrations of a mesophilic and a thermophilic reactor under varying conditions. In the mesophilic setup with process instability, propionic acid of the thermodynamic simulation represented experimental values well (7.8 g/L compared to 7.1 g/L), while the reference computation without a thermodynamic approach represented less than 1% of the experimentally observed concentration (0.01 g/L compared to 7.1 g/L). An investigation into mechanisms for process collapse under conditions with high acid concentrations ( > 8 g/L) but relatively stable pH (6.4) suggested the incorporation of an inhibition term by overall acid concentration for both methanogenic pathways.

## 1 Introduction

With a growing demand for alternative energy sources and waste management, a deeper understanding of anaerobic digestion offers important possibilities for advancing the controlled and calculable production of methane via biodegradation (Adler et al., 2014). As a multistage process underlying many dependencies on process conditions, modeling anaerobic digestion and codigestion requires the development of a complex reaction model that has been realized in different approaches; see, for example, the overview in Kelif Ibro et al. (2024). Of these models, the Anaerobic Digestion Model No. 1 (ADM1) is an established contemporary model that has been applied to various problems and used as groundwork for many extensions (Batstone and Keller, 2002; 2006; Kelif Ibro et al., 2024). Its kinetic rates for catalyzed reactions depend on reactant and microbial concentrations but not, in general, on product concentrations, in consequence allowing thermodynamically unfavorable reactions to take place that would not proceed due to the thermodynamic constraints. As these should be considered in cases where processes approach or exceed thermodynamic equilibrium, the purpose of this work was to develop a thermodynamic correction of ADM1 reaction rates and to examine and evaluate its impact using experimental data.

Additionally, Muha and Grillo (2012) demonstrated the importance of spatial resolution to compute realistic reaction rates for downflow reactors on a subset of ADM1 equations and provided a multiphase transport-reaction flow model based on the UG4 numerical framework. As thermodynamic conditions might vary in these cases, this work expands the model of Muha and Grillo (2012) to include all ADM1 reactions, which in turn are described using thermodynamically revised rates. As the aim of the current study is focused on the influence of thermodynamic considerations, the impact of the spatial aspect is reduced using assumptions of homogeneity and comparing to experimental data of continuously stirred tank reactors. Some of the work in this study was included in part in the Dissertation of Wittum (2025).

Based on the implementation by Muha and Grillo (2012), their previously limited reaction model is expanded, and a thermodynamic correction is introduced to modulate reaction rates as a function of thermodynamic conditions. The new model is run with and without the thermodynamic approach and compared to experimental data to investigate the impact of the introduced thermodynamic correction. Of special interest is the model behavior during unstable conditions in which reactions such as acid oxidations proceed close to thermodynamic equilibrium. These evaluations address the issue of identifying critical conditions for operating anaerobic digestion reactors under increasing loads.

The model extension developed here consists of three main components, namely (a) the biochemical pathway model for anaerobic digestion, (b) the thermodynamic constraints upon it, and (c) the underlying transport model for simulating spatially resolved reactors. For each aspect, previous models and research are available to use or build upon. These models are (a) the ADM1 as an established model in zero-dimensional, time-dependent frameworks (Batstone and Keller, 2002) including various suggestions for expansions, for example a syntrophic acetate oxidizing pathway by Gehring and Niedermayr (2016), (b) thermodynamic approaches to transform or modify the kinetic terms of individual chemical reactions in dependence on their associated free enthalpies (Hoh and Cord-Ruwisch, 1996; Jin and Bethke, 2007), and (c) the coupling of the reaction model with liquid transport processes in the multiphase flow model by Muha and Grillo (2012). All three aspects and the way they have been incorporated into the extended model will be described in Section 2, briefly touching on the base material. Section 3 presents results of multiple computations with the view of examining the impact of a thermodynamic approach and a model mechanism for lasting process collapse. These are followed by a discussion and succinct conclusion.

## 2 Model and methods

This work expands the implementation of Muha and Grillo (2012), which modeled only acetoclastic methanogenesis, with the intention of including additional ADM1 reactions in a multiphase flow model. The code was restructured and expanded to encompass the entire digestion process from substrate to methane and a spatially separate headspace; see Sections 2.1 and 2.3. A thermodynamic handling of reaction rates was added for the oxidation of valeric, butyric, propionic, and acetic acid as well as for acetoclastic and hydrogenotrophic methanogenesis; see Section 2.2. These expansions of the code were necessary in order to address the problem of process instability as a consequence of overfeeding a reactor. Computing units in the model are grams per liter for concentrations, excluding hydrogen ions, which are balanced for pH computation as moles per liter, gram-product per gram-processed reactant for reaction stoichiometries, and hours for time. The stoichiometry of some preconfigured chemical reactions is defined on a molar basis and identified as such, leading to a runtime conversion to mass-based units via associated molar mass values. The required input parameters are operating temperature $T_{\text{op}}$ in Kelvin, reactor fluid volume in liters, reactor type to determine transport mechanisms, reaction model to choose the set of chemical equations modeled, and simulation end time in hours. Additional input parameters, which are expedient to achieve a meaningful problem setup, include initial values, specifications of the feeding procedure, including an analysis of the substrate, or alternatively, in cases of downflow reactors, amounts of inflow with inflow analysis, and possibly digestate removal. Advanced input parameters concern individual model setup, for example, geometry, solver setup, chosen thermodynamic approach, or control over isolated model mechanisms, such as switching off inhibition terms. The output used for evaluation in this work includes average concentrations of valeric, butyric, propionic, and acetic acid in grams per liter and methane production rate in standard liters per hour and calculated average pH value. Additionally, concentrations of further intermediates and microbial groups are available for subsequent considerations but will not be presented in this work as a lack of experimental data limits their usefulness for compelling arguments.

### 2.1 Anaerobic digestion

**FIGURE 1:**
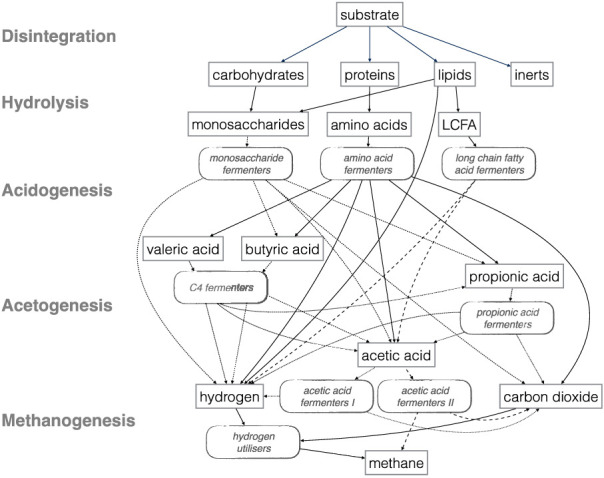
Schematic presentation of the basic reaction model, including model substances (boxes with sharp corners), fermentation pathways (arrows), and corresponding groups of microbial catalysts (boxes with rounded corners).

The ADM1 has been expanded to include a syntrophic pathway for the oxidation of acetic acid (Batstone and Keller, 2002; Gehring and Niedermayr, 2016). This leads to the reaction model presented in Figure 1 with model substances (boxes with square corners), functional groups of microorganisms (boxes with rounded corners), and reaction pathways (arrows). In accordance with the ADM1 model, kinetics for the disintegration of substrate and the hydrolysis of carbohydrates, proteins, and lipids were assumed to be first-order, whereas the subsequent steps of acidogenesis, acetogenesis, and methanogenesis were considered via Monod-type expressions modified by inhibition factors. As an extension to ADM1, other inhibition factors were included, these being an overall limit to microbial population similar to the implementation suggested in Muha and Grillo (2012), although only affecting microbial growth, and inhibition of methanogenesis by high overall acid concentration measured as acetic equivalence. In order to determine the stoichiometric factors of disintegration that are dependent on substrate composition, the procedure detailed in Koch and Lübken (2010) was applied with parameters of an extended Weender analysis, while all other stoichiometric factors were determined by associated chemical equations and do not vary depending on substrate. Equations used and potential differences to ADM1 are detailed in section A of the Supplementary Material.

Corresponding to the ADM1 approach, each modeled Monod-type reaction r considers one rate-limiting reactant j with the core reaction rate

$$R_{\text{core}}^{r}=k_{\text{m}}^{r}\frac{C_{j}^{r}C_{\text{mo}}^{r}}{K_{\text{M}}^{r}+C_{j}^{r}}\;\bar{I}$$ (1)

being equivalent to the reactant uptake rate, with the exception of hydrogenotrophic methanogenesis, where hydrogen and carbon dioxide uptake rates together equal the core reaction rate of Equation 1 above. Parameters $C_{j}^{r}$ and $C_{\text{mo}}^{r}$ designate the reactants and the corresponding group of catalyzing microorganisms concentrations respectively, while $k_{\text{m}}^{r}$ describes the maximum uptake rate, and $K_{\text{M}}^{r}$ is the Michaelis–Menten constant, representing the reactant concentration where half-maximal uptake is reached. The factor $\bar{I}$ is the product of all applied inhibition factors, which take values between 0, representing an almost complete kinetic standstill due to the associated inhibition mechanism, and 1, pointing to a negligible impact on the reaction rate under current conditions. Inhibition processes included for every Monod-type reaction rate are nutrient shortage via the inhibition term $I_{\text{IN}}$ depending on inorganic nitrogen concentration and pH-dependent inhibition via $I_{\text{pH}}$ . Degradation of valeric and butyric acid is catalyzed by the same microbial group leading to competitive uptake of these two reactants, represented by including the inhibition terms $I_{\text{b/v}}$ and $I_{\text{v/b}}$ in the respective core reaction rates, while acetoclastic methanogenesis may be inhibited by high ammonia concentrations through $I_{NH_{3}}$ . Inhibition by high hydrogen concentrations is considered via $I_{\text{H}2}$ in the degradation of long-chain fatty acids, which in this model does not underlie further thermodynamic constraints, while for acetogenic and methanogenic reactions, inhibition by hydrogen is assumed to be covered by taking thermodynamic limits into account. Table 1 contains an overview of all inhibition terms with further information on their expressions where $C_{i}$ describes the concentration of substance i in units gram per liter, $S_{{\text{H}}^{+}}$ is the concentration of hydrogen ions in moles per liter, and $K_{i}$ is the given inhibition constant in corresponding units. It includes the growth inhibition term $I_{\text{mo}}$ for microbial groups based on Muha and Grillo (2012), along with the inhibition of methanogenic pathways via $I_{\text{acids}}$ as extensions to ADM1 inhibition terms following the earlier work of Angelidaki et al. (1999). The latter is dependent on acetic equivalent acid concentrations and has been introduced to evaluate model behavior in cases of high total acidity under relatively neutral pH values. Unlike the original ADM1 approach for modeling pH inhibition by a discontinuous term, the expression presented in Table 1 was suggested by Siegrist and Vogt (2002) and subsequently by Rosen and Vrecko (2006) and Jeppsson and Rosen (2006). The inhibition constant $K_{\text{pH}}$ and the factor n are computed using predefined upper and lower limits ${pH}_{\text{UL}}$ , ${pH}_{\text{LL}}$ as.

$$K_{\text{pH}}=10^{-\frac{{pH}_{\text{LL}}+{pH}_{\text{UL}}}{2}},$$

$$n=\frac{3.0}{{pH}_{\text{UL}}-{pH}_{\text{LL}}}.$$

**TABLE 1:** Overview of inhibition terms considered in this work with a short description of the modeled mechanism, possible restrictions, and main reference in the literature.

Inhibition term	Description	Restriction	Reference
$I_{\text{pH}}=\frac{K_{\text{pH}}^{n}}{S_{H^{+}}^{n}+K_{\text{pH}}^{n}}$	pH inhibition for low pH values	​	b
$I_{\text{IN}}=\frac{C_{\text{IN}}}{K_{\text{IN}}+C_{\text{IN}}}$	Inhibition for $C_{\text{IN}}$ approaching zero	​	c
$I_{NH_{3}}=\frac{K_{NH_{3}}}{K_{NH_{3}}+C_{NH_{3}}}$	Inhibition caused by free ammonia	Only acetoclastic methanogens	c
$I_{H_{2}}=\frac{K_{H_{2}}}{K_{H_{2}}+C_{H_{2}}}$	Inhibition caused by hydrogen	Only long-chain fatty acid (LCFA) fermenters, others: thermodynamic limit	c
$I_{\text{b/v}}=\frac{C_{\text{b/v}}}{C_{\text{b/v}}+C_{\text{v/b}}}$	Competition by butyric (b) or valeric (v) acid	Only valeric/butyric fermenters, respectively	c
$I_{\text{mo}}=1-\frac{C_{\text{mo}}}{K_{\text{mo},\text{ max}}}$	Inhibition of microbial growth at dense populations	​	d
$I_{\text{acids}}=\frac{K_{\text{acids}}}{K_{\text{acids}}+C_{\text{acids}}}$	Inhibition of methanogens through overall acidity	Only acetoclastic (and hydrogenotrophic)^a^ methanogenesis	e

^a^ Calculations considering $I_{\text{acids}}$ for the hydrogenotrophic pathway are identified explicitly.

^b^ Rosen and Vrecko (2006); Jeppsson and Rosen (2006).

^c^ Batstone and Keller (2002).

^d^ Muha and Grillo (2012).

^e^ Angelidaki et al. (1999).

Growth of catalyzing microorganisms is driven by reactant uptake, where each microbial group uses up to a fraction of $Y_{\text{mo}}^{r}$ of the corresponding core reaction rate for growth, modulated by $I_{\text{mo}}$ , thus taking the form

$$R_{\text{mo}}^{r}=Y_{\text{mo}}^{r}I_{\text{mo}}R_{\text{core}}^{r}.$$ (2)

The remaining fraction $1-Y_{\text{mo}}^{r}I_{\text{mo}}$ of the core reaction rate flows into product generation, where each product k is produced by a reaction r according to its mass-based stoichiometric factor $\mu_{k}^{r}$ , yielding

$$R_{k}^{r}=\mu_{k}^{r}\left(1-Y_{\text{mo}}^{r}I_{\text{mo}}\right)R_{\text{core}}^{r}.$$

In order to compute overall source and sink terms $R_{i}^{\Sigma }$ according to the reaction model for each substance i , contributions from all possible reactions r must be considered via

$$R_{i}^{\Sigma }=\underset{r}{\sum }R_{i}^{r},$$ (3)

where i acts either as reactant or product, leading to negative or positive rates, respectively. For microbial groups, growth and decay must be considered with growth being dependent on reactions rates according to Equation 2 and a steady microbial death rate $k_{\text{mo}}^{\text{dec}}$ , leading to

$$R_{\text{mo}}^{\Sigma }=\underset{r}{\sum }R_{\text{mo}}^{r}-k_{\text{mo}}^{\text{dec}}C_{\text{mo}}.$$ (4)

As microbial groups are assigned carbon content $f^{CO_{2}}$, their growth and decay affect carbon dioxide concentration directly, so that $R_{CO_{2}}^{\Sigma }$ is computed via

$$R_{CO_{2}}^{\Sigma }=\underset{r}{\sum }\left(R_{CO_{2}}^{r}-f_{\text{mo}}^{CO_{2}}R_{\text{mo}}^{r}\right)+\underset{\text{mo}}{\sum }f_{\text{mo}}^{CO_{2}}k_{\text{mo}}^{\text{dec}}C_{\text{mo}}.$$ (5)

Nitrogen as the sum of $NH_{3}$ and $NH_{4}N$ is not included in the reaction model as a reactant or product, but all microbial groups and some additional model substances such as proteins and amino acids are ascribed a nitrogen content $f_{i}^{\text{N}}$ , leading to changes in overall inorganic nitrogen concentration via the effective reaction term

$$R_{\text{N}}^{\Sigma }=\underset{i}{\sum }f_{i}^{\text{N}}R_{i}^{\Sigma }.$$ (6)

Due to differences in model units, kinetic parameters and some inhibition constants provided by Batstone and Keller (2002) and Jeppsson and Rosen (2006) must be transformed. Computed values used for the kinetic parameters $k_{m}^{r}$ , $K_{M}^{r}$ , $Y_{\text{mo}}^{r}$ , and $k_{\text{mo}}^{\text{dec}}$ and for inhibition constants $K_{\text{IN}}$ , $K_{NH_{3}}$ , $K_{H_{2}}$ , $K_{\text{mo},\text{ max}}$ , and $K_{\text{acids}}$ , are found in the Supplementary Material.

For hydrogen ions, a different approach is taken by ensuring the charge balance is met instead of compiling source and sink terms, corresponding to the method employed in the ADM1. The concentration of hydrogen ions is found by solving the charge balance equation

$$S_{{\text{H}}^{+}}=\underset{i\in \text{acids}}{\sum }\frac{K_{s,\;i}\cdot C_{i}}{M_{i}\;\left(K_{s,\;i}+S_{{\text{H}}^{+}}\right)}-\underset{i\in bases}{\sum }\frac{S_{{\text{H}}^{+}}\cdot C_{i}}{M_{i}\;\left(K_{s,\;i}+S_{{\text{H}}^{+}}\right)}+\frac{K_{\text{w}}}{S_{{\text{H}}^{+}}}+S_{\text{offset}},$$

with model substance concentrations $C_{i}$ , the offset $S_{\text{offset}}$ , acid dissociation constants $pK_{s}$ , self-ionization constant of water $pK_{w}$ , and molar masses $M_{i}$ . The sum over all acids includes valeric, butyric, propionic, and acetic acids and hydrogen carbonate, while inorganic nitrogen is the only base considered in this model. The offset $S_{\text{offset}}$ comprises additional anions and cations and is computed using reactor starting conditions, including acid and nitrogen concentrations and initial pH.

### 2.2 Thermodynamic approach

Included within elaborative discussions regarding ADM1 development, the thermodynamic approach of Hoh and Cord-Ruwisch (1996) is mentioned as an alternative to model inhibition due to hydrogen accumulation (Batstone and Keller, 2002). It is based on thermodynamic considerations that prohibit positive reaction rates in cases of unfavorable energy balance; that is, a reaction will only take place if the catalyzing microbes can benefit energetically. This thermodynamic limitation is not exclusive to hydrogen. It applies to any product involved in the reaction, meaning that an accumulation of products may result in thermodynamically unfavorable conditions that affect the overall process. In systems of constant pressure and volume, the energy balance of a chemical reaction can be expressed via changes in Gibbs free energy ΔG using

ΔG=ΔH−TΔS,

with enthalpy change ΔH , entropy change ΔS , and temperature T . If the starting point Gibbs free energy is larger than that of the end state, energy is released by the reaction, which may occur spontaneously, and ΔG<0 holds. For ΔG>0 , energy must be added to the system to proceed, and the reaction in question will not occur spontaneously. With respect to energy benefit, the reverse reaction would be preferred thermodynamically, although in general, the energetic possibility of any reaction does not allow drawing conclusions about either it actually taking place or the associated reaction speed. Under standard conditions for all reactants and products, the resulting standard Gibbs free energy ΔG° is directly linked to the equation’s equilibrium constant K via

ΔG°=−R T⁡ln⁡K,

and if reactant and product activities, or in cases of highly diluted solutions their concentrations, are not equal to their thermodynamic standard states, the resulting Gibbs free energy can be expressed via the associated reaction quotient Γ , yielding

$$\Delta G=R\;T\ln\left[\Gamma /K\right].$$

Hoh and Cord-Ruwisch (1996) derive a thermodynamically modulated kinetic term based on the reversible model by Haldane with some assumptions that transforms the core reaction term of Equation 1 for one limiting reactant j to

$$R_{\text{core},\text{ transform}}^{r}=k_{\text{m}}^{r}\frac{C_{j}^{r}\;\left(1-\frac{\Gamma^{r}}{K^{r}}\right)}{K_{\text{M}}^{r}+C_{j}^{r}\;\left(1+k_{r}\frac{\Gamma^{r}}{K^{r}}\right)}\;C_{\text{mo}}^{r}\bar{I},$$ (7)

with the factor $k_{r}$ controlling the reverse reaction rate compared to the forward rate (Hoh and Cord-Ruwisch, 1996). Another approach with some expansions to include an averaged stoichiometric factor χ for non-elementary steps in the reaction progress and a microbial maintenance term $\Delta G_{\text{mo}}$ was introduced by Jin and Bethke (2007) using Monod-type kinetics that are multiplied by a thermodynamic factor F similar to the inhibition terms. With the simplifying assumptions of χ=1 and $\Delta G_{\text{mo}}\approx 0$ , the thermodynamic factor takes the form

$$F=\left\{\begin{array}{cc} 1-\exp\left(-\frac{\Delta G^{r}}{RT}\right) & \text{if }\Delta G^{r}>0\\ 0 & \text{else} \end{array}\right.$$

and leads to a core reaction rate based on Equation 1 of

$$R_{\text{core},\text{ factor}}^{r}=k_{\text{m}}^{r}\frac{C_{j}^{r}}{K_{\text{M}}^{r}+C_{j}^{r}}\;C_{\text{mo}}^{r}\;\left(1-\frac{\Gamma^{r}}{K^{r}}\right)\bar{I}$$ (8)

in thermodynamically favorable conditions, that is, Γ/K<1 . With the assumptions made for the Jin and Bethke (2007) approach, it yields the same reaction rate as the Hoh and Cord-Ruwisch (1996) model for a reverse factor $k_{r}=0$ and explicitly suppressed reverse reactions. The model presented in this work supports both approaches and provides the possibility of allowing reverse reactions via a damping factor if the factorial approach by Jin and Bethke (2007) is chosen.

In order to compute the thermodynamic term $\Gamma^{r}/K^{r}$ for a reaction r , the standard Gibbs free energy must be known to find the equilibrium constant $K^{r}$ . For the reaction quotient $\Gamma^{r}$ , stoichiometric coefficients and concentrations specific in time and space for all participating reactants and products must be determined. For many reactions considered in the model, tabulated values of corresponding Gibbs free energies under standard conditions exist, for example, by Thauer et al. (1977), Hanselmann (1991), and Schink (1997), or values can be extrapolated from values of the individual reactants and products, for example, from Landolt and Börnstein (1971), Lechner (1992), or Hanselmann (1991) (Supplementary Tables A.1 and A.2). The tabulated values apply to temperatures of 25°C and per default to pH 0, if not indicated otherwise. In the case of gases, they usually refer to their gaseous state. Depending on the characteristics of modeled substance concentrations, tabulated values may not be suitable for direct comparisons with the reaction quotient $\Gamma^{r}$ being provided in reference to model conditions, that is, gases in aqueous states or acids depending on the actual pH value. Therefore, the standard Gibbs free energies of all model reactions that are treated thermodynamically in this work were derived to represent reactants and products in their aqueous state and for non-ionized acids to avoid dependence on reference pH values using tabulated data and methods from Hanselmann (1991). Table 2 contains the obtained associated standard Gibbs free energies for all thermodynamically modeled reactions used in this work.

**TABLE 2:** Overview of all thermodynamically treated reactions with corresponding standard Gibbs free energies for unionized acids and gases in their aqueous states, as derived using tabulated values from Hanselmann (1991).

Reaction	$\Delta G^{r,◦}\;\left[\frac{kJ}{mol}\right]$
Oxidation of valeric acid ${CH}_{3}{\left({CH}_{2}\right)}_{3}$ COOH +2 $H_{2}{O\to CH}_{3}{CH}_{2}$ COOH + ${CH}_{3}$ COOH +2 $H_{2}$	95.8
Oxidation of butyric acid ${CH}_{3}{\left({CH}_{2}\right)}_{2}$ COOH +2 $H_{2}$ O $\to {CH}_{3}$ COOH +2 $H_{2}$	96.4
Oxidation of propionic acid ${CH}_{3}{CH}_{2}$ COOH +2 $H_{2}{O\to CH}_{3}$ COOH + ${CO}_{2}$ + 3 $H_{2}$	133.5
Acetoclastic methanogenesis ${CH}_{3}$ ${COOH\to CH}_{4}$ + ${CO}_{2}$	−23.8
Oxidation of acetic acid ${CH}_{3}$ COOH +2 $H_{2}$ O $\to {CO}_{2}$ + 4 $H_{2}$	169.2
Hydrogenotrophic methanogenesis ${CO}_{2}$ + 4 $H_{2}$ $\to {CH}_{4}$ + 2 $H_{2}$ O	−193.0

Computations with $R_{\text{core},\text{ transform}}^{r}$ according to Equation 7 are performed using $k_{r}=1$ for all reactions in Table 2, while computations with $R_{\text{core},\text{ factor}}^{r}$ according to Equation 1 allow a fully reversible methanogenesis, a damped reversal of propionic acid oxidation, and no reverse reactions for the oxidation of butyric and valeric acid (i.e. the backward reaction rate is set to zero if thermodynamic conditions would favor the reverse over the forward reaction).

### 2.3 Transport processes and gas phase modeling

The model follows the two-phase flow approach introduced by Muha and Grillo (2012) for a single reaction and which couples Monod-type kinetics with transport processes via a set of convection–diffusion equations. In addition to the two-phase flow model, Muha (2012) presents a three-phase flow model but prioritized the two-phase flow model due to the high computational costs of a fully coupled approach caused by time-scale differences. This work expands the groundwork laid by Muha (2012) and Muha and Grillo (2012) with the two-phase flow model by modeling gas transport via separate equations and factoring in the free and dissolved states, instead of including gaseous substances directly in the multiphase model. To account for the importance of product removal, including gases and especially hydrogen, from the digestate under thermodynamic considerations, the model of this work includes a geometrically separated headspace. Thus, this model uses reactor geometries characterized by two main subsets, one designated for the digestate, which corresponds to the main geometric subset of Muha and Grillo (2012), accommodating all anaerobic digestion model equations, and one as headspace, containing exclusively free gases with no participation in fluid flow or the biochemical reaction model. To avoid discontinuous solutions for the concentrations $C_{i}^{g}$ at the interface between digestate and headspace, continuous functions

$$u_{i}^{g}=K_{i}^{j}C_{i}^{g,j}$$

are introduced for all gases g with partition coefficients $K_{i}^{j}$ , satisfying

$$\begin{array}{lll} K_{i}^{\text{aqueous}}C_{i}^{g,\text{ aqueous}} & =K_{i}^{\text{gas}}C_{i}^{g,\text{ gas}} & \;\text{on the interface}. \end{array}$$

Applying Henry’s law to the partition coefficient ratio and choosing $K_{i}^{\text{aqueous}}=1$ yields

$$\frac{\partial }{\partial t}\left[u_{i}^{g}\right]-\nabla \cdot \left[D_{i}^{l}\;\nabla u_{i}^{g}-\vec{v}\;u_{i}^{g}\right]=R_{i}^{\text{source/sink}}\;\text{in the digestate},$$ (9)

$$\frac{\partial }{\partial t}\left[\frac{1}{H_{i}^{\text{cc}}}u_{i}^{g}\right]-\nabla \cdot \left[\frac{D_{i}^{g}}{H_{i}^{\text{cc}}}\;\nabla u_{i}^{g}\right]=R_{i}^{\text{source/sink}}\;\text{in the headspace},$$ (10)

with diffusion coefficients $D_{i}^{l}$ in the liquid digestate and $D_{i}^{g}$ in the headspace as equations for model substances assigned to the gas phase. The treatment of carbon dioxide presents an exception as its dissolved form dissociates quickly toward an equilibrium distribution signified by $K_{{\text{CO}}_{2}}$ with hydrogen carbonate. Maintaining $K_{{\text{CO}}_{2}}^{\text{aqueous}}=1$ , the partition coefficient $K_{{\text{CO}}_{2}}^{\text{gas}}$ transforms in the following way

$$\;\frac{1}{H_{{\text{CO}}_{2}}^{\text{cc}}}\to \frac{S_{{\text{H}}^{+}}}{K_{{\text{CO}}_{2}}+S_{{\text{H}}^{+}}}\cdot \frac{1}{H_{{\text{CO}}_{2}}^{\text{cc}}}.$$

Source and sink terms listed on the right-hand side of Equation 9 and analogically in the respective equations for liquid and solid phase substances refer to processes taking place in the digestate and contain all reaction rates according to Equations 3–6 and reactor interactions such as feeding and digestate removal. These processes play no direct role in Equation 10, which is only applicable in the reactor headspace. There, only gas removal from the headspace in cases where headspace pressure surpasses a reference value, chosen as atmospheric pressure, is modeled.

### 2.4 Model application

Model implementation was carried out using the UG4 numerical framework, which supports the discretization and solving of numerically complex systems of partial differential equations in structured and unstructured grids (Vogel and Reiter, 2014; Reiter and Vogel, 2014; Vogel, 2014). A computational grid, representing a 2D rectangular reactor geometry with a length of 10.07 dm and a height of 2.0 dm, was created using ProMesh (Reiter and Vogel, 2014). Grid elements were assigned to different subsets, either a 1D boundary subset or one of two area subsets. The latter represent the reactor space for digestate (height of 1.45 dm) and the headspace for free gases. The 2D grid models a projection of the full 3D reactor that represents the relevant processes due to the assumed homogeneity. Input and output values are scaled based on the 2D grid and a user-specified volume for the full reactor. The grid was refined at the start of each computation in UG4, and a convergence analysis was performed beforehand to ensure that observed effects concerning process collapse are not the result of insufficient refinement. Time discretization was performed using the linearly implicit extrapolation scheme Limex with adaptive time step sizes, using an initial step size of 0.012 h, maximum step size of 1 h, and minimum step size of 0.00001 h. For spatial discretization, the finite volume method supported by UG4’s ConvectionDiffusion plugin was used (Vogel, 2014). A geometric multigrid scheme with three pre- and post-smoothing steps, respectively, in a V-cycle was accelerated by a BiCGSTAB method. For details on the numerical solver methods, see, for example, Hackbusch (1985). For all outer boundaries, no-flow Neumann conditions are used, indicating that no reactor content is added or lost via these boundaries. Following assumptions of homogeneity, the reactor feeding, digestate removal, and the pressure-dependent gas removal from the headspace are modeled via reaction terms; therefore, no transport across grid boundaries is needed.

Kinetic parameters correspond to ADM1 values as used in the benchmark implementations of Rosen and Vrecko (2006) and Jeppsson and Rosen (2006), with the exception of those pertaining to the oxidation of acetic acid, which was not originally included in the ADM1, and are assigned values based on Gehring and Niedermayr (2016). As these kinetic parameters are given in units of oxygen demand, they are first transformed into the applicable model units, yielding the values summarized in Supplementary Table S1. The same applies to inhibition constants included in the ADM1, which are provided in their transformed units in Section B of the Supplementary Material alongside the inhibition constants of additionally implemented mechanisms. Physicochemical constants used in the model, such as acid dissociation constants, Henry coefficients, and diffusion coefficients, are provided in Section C of the Supplementary Material.

The model has been applied to experimental data acquired during the Modisto project (Lebuhn et al., 2021), mainly in order to evaluate the effect of the thermodynamic approach and secondarily to gain insight into mechanisms causal to permanent process standstill. For the mesophilic reactor setup, the operating temperature of the model reactor was set to 311.15 K, and the computation was run from 0 h to 4080 h of simulated time, corresponding to the runtime of the mesophilic reactor in the Modisto project (Lebuhn et al., 2021). Analogously, the thermophilic reactor’s operating temperature was set to 325.15 K, and this computation was run up to 3500 h of simulated time. These timeframes relate to the experimental settings for the mesophilic and thermophilic reactors during the second experimental series of the Modisto project; see Sections II.1.1.8.4 and II.1.1.8.5 in Lebuhn et al. (2021), respectively.

**FIGURE 2:**
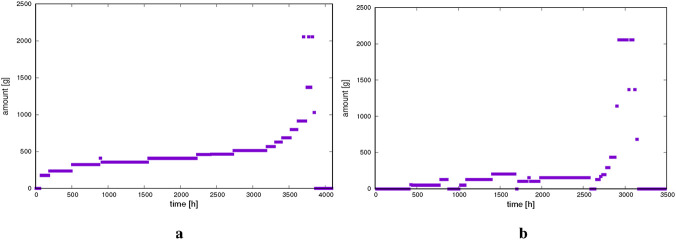
Profiles for the reactor feeding amounts used as model input with data points every 24 h. Both model setups show a similar tendency with lower initial values, moderate reactor input during an extended intermediate interval, and quickly increased amounts near the end of reactor runtime. **(a)** Mesophilic setup. **(b)** Thermophilic setup.

The feeding procedures for both model setups are presented in Figure 2 showing the amount of maize silage fed to the 40 L reactors. Each feeding event adds to the model substrate and other model substances according to the composition detailed in Table 3 and the fed amount of maize silage. Feeding amounts in the mesophilic setup (see Figure 2a) were increased incrementally with a sharp increase at approximately 3500 h shortly before feeding was suspended entirely. Between 1000 h and 3000 h of simulated time, the amount of maize silage fed to the reactor stayed relatively constant and indicates the stable reactor phase, while the high amounts after 3500 h are meant to provoke reactor instability and a collapse of the digestion processes; see Lebuhn et al. (2021) for additional information. In the thermophilic setup, a similar approach was chosen, although the occurrence of an unintentional process instability during the intended stable phase required adjustments to the feeding procedure and led to a generally lower input; see Figure 2b. Following the previously described procedure, final process instability and collapse were initiated by intense overfeeding. Both model setups use an identical reactor volume of 40 L, which was maintained via digestate removal in the experiment. The corresponding data for model input (that is, digestate removal in liters) were provided by Rüppel (2019). Initial values for both model reactor setups are listed in Table 4.

**TABLE 3:** Overview of model feedstock composition. Values are calculated based on an analysis of maize silage fed to the experimental reactors. See Section A in the Supplementary Material for the stoichiometric parameters.

​	Value
Quantity	Model feedstock
Dry matter [%]	41.4
Organic DM [%]	97.12
Carbohydrates [%]	74.6
Proteins [%]	8.3
Lipids [%]	3.5
Acetic acid [g/L]	11.74
Propionic acid [g/L]	0.63
Butyric acid [g/L]	0.11
Valeric acid [g/L]	0.06
Carbon dioxide [g/L]	1.67
Nitrogen [g/L]	0.42

**TABLE 4:** Overview of initial conditions for both reactor setups. Values in the table starting from pH to nitrogen are taken from experimental measurements, while values for substrate to long-chain fatty acids (LCFAs) are based on short model runs under stable conditions. Microbial concentrations of 1.0 g/L are assumed as a default setting. The same holds for the near-zero value assumed for propionic and valeric acid in the mesophilic setup.

​	Values for different setups	
Quantity	Mesophilic setup	Thermophilic setup
pH	7.97	8.38
Dry matter [%]	9.13	13.0
Organic DM [%]	73.77	79.0
Acetic acid [g/L]	0.3758	2.3302
Propionic acid [g/L]	E−7	0.2418
Butyric acid [g/L]	0.01	0.1947
Valeric acid [g/L]	E−7	0.2630
Carbon dioxide [g/L]	10.5	19.0
Nitrogen [g/L]	3.352	4.2
Substrate [g/L]	5.7	1.7
Carbohydrates [g/L]	4.7	1.41
Proteins [g/L]	1.3	1.1
Lipids [g/L]	1.1	0.34
Monosaccharides [g/L]	0.004	0.006
Amino acids [g/L]	0.002	0.001
LCFA [g/L]	0.004	0.001
Microbial groups [g/L]	1.0	1.0

Model computations were performed using the settings presented above, and the results were compared to experimental data with a focus on methane production and acid concentrations. Comparisons with experimental findings use the original data on which Figures 9–12 of Lebuhn et al. (2021) are based, although the data have been transformed in order to match the units of this model. Additional information was provided by Munk (2019), among which the pH values during process instability and collapse are of special interest. In the mesophilic case, pH reached a minimum of 6.41; in a thermophilic reactor, pH reached a minimum of 5.37. For evaluating the thermodynamic approach, both model reactor setups were run with and without thermodynamic reaction rates to compare respective results. Mechanisms for process collapse were investigated using the mesophilic setup as experimentally observed pH stayed relatively stable despite high acid concentrations. It should be noted that all computations were run with active inhibition by pH, nitrogen, etc., according to Table 1.

### 2.5 Evaluation and sensitivity analysis

Model results using different setups are compared to experimental data with a focus on methane production rate and acid concentrations. To evaluate the thermodynamic approach, the general profiles for these values are observed and judged according to the expected behavior based on experimental data. This includes low concentrations, especially of longer-chain fatty acids, during stable reactor phases, with increasing amounts during overfeeding and process collapse, indicated by a drop-off in methane production. In addition, quantitative comparisons are performed for the stable and the unstable reactor phase of the mesophilic setup using representative data points and acid concentration values shown in Table 5. These experimental values illustrate the overall behavior with acid concentrations, especially propionic acid, increasing in the experimental reactor during process instability.

**TABLE 5:** Overview of representative values for acid concentrations in the experimental reactor under mesophilic conditions. These data originate from the Modisto project (Lebuhn et al., 2021) and were obtained from Munk (2019).

​	Values for different setups			
​	Stable phase		Unstable phase	
Quantity	1255 h	2575 h	3877 h	3949 h
Acetic acid [g/L]	0.300	0.257	2.785	3.063
Propionic acid [g/L]	0.102	0.030	7.070	7.120
Butyric acid [g/L]	0.122	0.040	2.347	2.284
Valeric acid [g/L]	0.086	0.069	1.235	1.276

In order to gauge parameter dependencies, a local sensitivity analysis was performed for the kinetic parameters $k_{m}$ , $K_{M}$ , $k_{\text{dec}}$ , and $Y_{\text{mo}}$ and the parameter $K_{\text{mo},\text{ max}}$ for acetoclastic and hydrogenotrophic methanogenesis in the mesophilic reactor setup. Each parameter was varied individually by ± 10, ± 20, and ± 30%, and for each parameter variation, the least squares target function

$$f\left(X_{\text{exp}},X_{\text{sim}}\right)=\frac{1}{N_{\text{exp}}}\overset{N\text{exp}}{\underset{i}{\sum }}{\left(X_{\text{exp}}^{i}-X_{\text{sim}}^{i}\right)}^{2}$$ (11)

was evaluated for $N_{\text{exp}}$ experimental data points $X_{\text{exp}}^{i}$ and the corresponding simulation results $X_{\text{sim}}^{i}$ . The set of experimental data points $X_{\text{exp}}^{i}$ considered comprises a total of 18 values for acetic and propionic acid concentrations, and simulation results were evaluated at corresponding points in time, generally by linear interpolation between adjacent datasets.

Differences in simulation results induced by input variation are subsequently computed relative to the reference simulation, i.e., the fraction

$$\frac{f_{i}\left(X_{\text{exp}},X_{\text{sim}}\right)-f_{\text{ref}}\left(X_{\text{exp}},X_{\text{sim}}\right)}{f_{\text{ref}}\left(X_{\text{exp}},X_{\text{sim}}\right)},$$ (12)

is determined for all input variations i . The value of $f_{\text{ref}}\left(X_{\text{exp}},X_{\text{sim}}\right)$ is computed by applying Equation 11 to the results of the mesophilic model setup with thermodynamic correction using the unit-transformed ADM1 input values of Supplementary Table S1. A negative relative change in the target function $f\left(X_{\text{exp}},X_{\text{sim}}\right)$ , as defined by Equation 12, indicates a better agreement with experimental data compared to the reference simulation, and positive values indicate a larger deviation from them. Parameters that strongly influence results can be identified by a larger relative change of $f\left(X_{\text{exp}},X_{\text{sim}}\right)$ .

## 3 Results

### 3.1 Influence of thermodynamics

**FIGURE 3:**
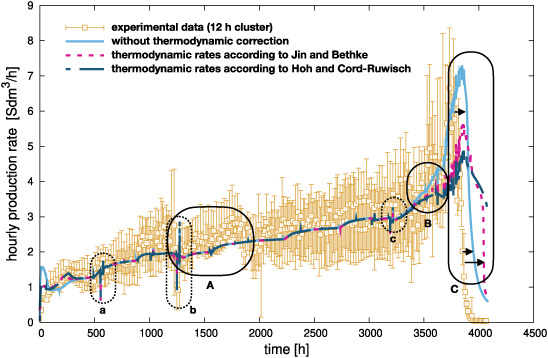
Comparison of methane production rates in standard cubic decimeters per hour for computations using two different or no thermodynamic approaches with clustered experimental data. Area A displays discrepancies between all computational rates and the experimental average due to the addition of digestate into the experimental reactor, which was not included in the simulation. Area B marks emerging differences between computations with or without thermodynamically modified reaction rates in the process of reactor overfeeding, while significantly different profiles are apparent in Area C with all computational curves decreasing later in time compared to the experimental data. The interspersed oscillations, for example, in the areas marked with dotted lines as a, b, and c, correlate with larger amounts of digestate removed. acetic acid propionic acid butyric acid valeric acid.

The first series of results for the mesophilic setup compares the computation without thermodynamic reaction rates, although including hydrogen inhibition at the oxidative reactions, with two computations using a thermodynamic approach, either according to Equation 7 or Equation 8, corresponding to the simple approach by Hoh and Cord-Ruwisch (1996) or Jin and Bethke (2007), respectively. Figure 3 displays methane production rate profiles for all three computations using simple polygonal chains via *gnuplot* and experimental data. For the latter, available hourly values were clustered, averaged, and provided with minimum to maximum ranges in 12 h intervals. Corresponding to reactor input via feeding, the stable reactor operation time window ranges between 500 h and 3000 h, during which period all computations yield comparable methane production rates independent of thermodynamic approach. Additionally, the computed results correspond well to the experimental data, except the area marked with A in Figure 3. Due to a relatively large amount of digestate taken from the experimental reactor for testing purposes, the volume deficit of this reactor was compensated by adding digestate from a different mesophilic reactor. The simulations neglected this addition of organic mass into the reactor; therefore, the subsequent underestimation of the methane production rate is not surprising. Starting between 3000 h and 3500 h, overfeeding of the reactor was initiated, leading at first to an increase in produced methane and finally, in the experimental data, to a sudden collapse of methane production, indicating adverse process conditions. During these events, differences between different thermodynamic approaches become apparent; see areas B and C in Figure 3. In general, the profile generated without including thermodynamic reaction rates exhibits a closer affinity to the experimental data, observed, for example, in the increase and the steep fall of the methane production rate, although the latter occurs at a temporal distance from the experimental decrease. Further analysis shows that the production decrease in all computations happens only after feeding has stopped, pointing to a lack of organic material as opposed to process collapse as the origin for the decrease in the methane production rate.

**FIGURE 4:**
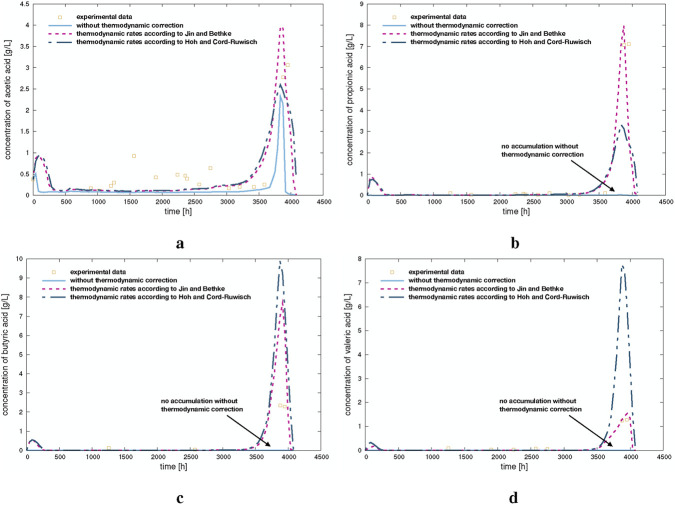
Comparison of acid concentration profiles for two computations with different thermodynamic approaches, one computation without thermodynamic correction, and the experimental data of Lebuhn et al. (2021). Black arrows indicate the low values for longer-chain acids in the computation without thermodynamic considerations. **(a)** Acetic acid. **(b)** Propionic acid. **(c)** Butyric acid. **(d)** Valeric acid.

Results for the acid concentration profiles are provided in Figure 4 for acetic, propionic, butyric, and valeric acid for all three computations in comparison with experimental data, as shown in Figure 10 in Lebuhn et al. (2021). In general, the concentrations of acetic and, to a lesser degree, of propionic acid are underestimated by the computations during stable reactor phases while overall reflecting stable conditions. A quantitative comparison, using the data presented in Tables 5, 6, confirms this. At t = 1255 h, the values in the computation with thermodynamic correction represent 35.56% of acetic, 14.43% of propionic, 4.22% of butyric, and 2.03% of valeric acid measured experimentally at these time points. However, for butyric and valeric acid, only a small number of measurements during the stable phase are recorded with values above zero for these long-chain acids, assuming that concentrations were too low to be determined. For the second sample during the stable reactor phase at t = 2575 h, the simulation results yield 57.53% of acetic, 100.09% of propionic, 20.59% of butyric, and 3.65% of valeric acid concentrations. For both time points, the computation without thermodynamic correction yields even lower acid concentrations. Overall, the low acid concentrations during the stable process are reflected by both computations, and the stable phase results propose no significant superiority of the thermodynamic approach. This changes when examining the model behavior that results from overfeeding.

**TABLE 6:** Results for acid concentrations at simulation times corresponding to the experimental data points in Table 5. The simulation results are from the mesophilic reactor setup, either without thermodynamic correction or with the approach according to Jin and Bethke (2007). Additionally, the concentration maxima are presented for both model setups.

​	Values for different setups and timepoints				
​	Stable phase		Unstable phase		
Quantity	1255 h	2575 h	3877 h	3949 h	Max
Without thermodynamic correction					
Acetic acid [g/L]	0.074	0.075	1.913	0.042	2.806
Propionic acid [g/L]	0.005	0.005	0.012	0.002	0.021
Butyric acid [g/L]	0.004	0.004	0.008	0.002	0.012
Valeric acid [g/L]	0.001	0.002	0.003	0.001	0.004
With thermodynamic correction					
Acetic acid [g/L]	0.107	0.148	3.858	2.016	4.183
Propionic acid [g/L]	0.015	0.030	7.851	2.502	8.169
Butyric acid [g/L]	0.005	0.008	7.034	6.456	7.794
Valeric acid [g/L]	0.002	0.003	1.290	1.522	1.594

During process instability, all acid concentrations are expected to increase significantly, judging from the experimental data; see datasets for t = 3877 h and t = 3949 h in Table 5, with values reaching 1.2 g/L for valeric acid and above 7.1 g/L for propionic acid. In the computation without the thermodynamic approach, only acetic acid reaches comparable concentrations with a maximum value of 2.8 g/L, while concentrations of propionic, butyric, and valeric acid with maxima below 0.01 g/L are far from matching experimental values. Expressed quantitatively, the simulation results at t = 3877 h represent 68.92% of acetic, 0.18% of propionic, 0.34% of butyric, and 0.26% of valeric acid concentrations. At t = 3949 h, they represent only small fractions of the experimental values, with respective percentages of 1.36%, 0.03%, 0.10%, and 0.10%. The computations with the thermodynamic approach correspond much better to experimentally observed behavior, reaching concentrations between 1.3 g/L for valeric and up to 7.9 g/L for propionic acid at t = 3877 h. For this dataset, the computation with a thermodynamic approach based on Jin and Bethke (2007) overestimates experimental acid concentrations by 39.01% for acetic, 11.04% for propionic, 199.98% for butyric, and 4.52% for valeric acid. At the later sample point, those concentrations, except for valeric acid, have decreased, and simulation results for acetic and propionic acid underestimate experimental values, representing 65.82% and 35.14% of experimentally observed acid concentration, respectively.

The described behavior can be gauged qualitatively from Figures 4a–d. Focusing on acetic acid (4a), all computations exhibit lower values during the stable phase and an increasing concentration during overfeeding. For the computation without a thermodynamic approach, the concentration profile corresponds relatively well to the experimental data with a sharp increase between 3500 h and 4000 h, although experimental values are not reached, while both thermodynamic computations exhibit a less abrupt increase that starts before 3500 h and lasts longer; see Figure 4a. Of these computations, the approach based on Jin and Bethke (2007) reaches and surpasses the experimental data points. Important differences between computations become apparent for the longer-chain acids. Without thermodynamic considerations, neither propionic, butyric, nor valeric acid concentration profiles exhibit the expected characteristic increase during process instability; see the black arrows in Figure 4. These accumulations of acids are only reproduced by simulations with a thermodynamic approach.

In contrast to experimental results, all increased acid concentrations decrease again. In the case of acetic acid in the non-thermodynamic computation, the decrease occurs even before the experimental values are reached, both in time and in amount, pointing to a process recovery instead of a collapse. The same behavior can be observed for propionic, butyric, and valeric acids in both thermodynamic computations. Their concentrations increase to values explicitly indicative of process instability but fall again after feeding has stopped, pointing to a recovery of the previously unstable process.

**FIGURE 5:**
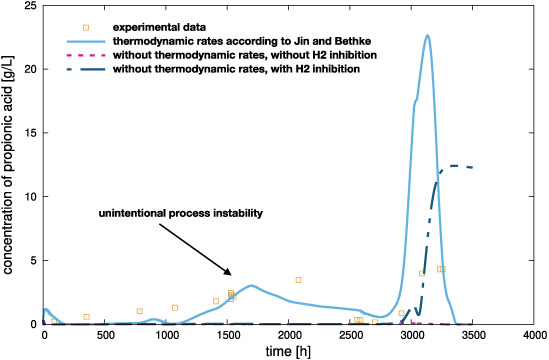
Comparison of propionic acid concentration profiles for two computations without the thermodynamic approach, either including or disregarding hydrogen inhibition, one computation with the thermodynamic approach, and the experimental data of Lebuhn et al. (2021) for the thermophilic setup.

Complementing these results, an additional set of computations with and without thermodynamic reaction rates is compared, based on the thermophilic dataset, with a supplementary test of disregarding the hydrogen inhibition term in the non-thermodynamic computation. Figure 5 contains the profiles of propionic acid for three different computational setups, indicating that the provoked final process instability can be observed with and without the thermodynamic approach as long as hydrogen inhibition is included for the setup where no thermodynamic correction is considered. In this case, acid profiles behave as would be expected during process collapse. However, the unintentional reactor instability demonstrated by an increase in longer-chain acids is only reproduced by the thermodynamic computation, as shown representatively for propionic acid in Figure 5 (black arrow). Taking the highest value for propionic acid in the experimental data from the cluster at approximately t = 1530 h, the thermodynamic computation yields 82.91% of the experimentally observed concentration, while both computations without a thermodynamic approach yield far less, with 0.66% without hydrogen inhibition and 1.37% with hydrogen inhibition. The experimental and simulated pH values (not shown) stayed above 8.0 for the entire run up until 2800–3000 h, indicating that this unintentional instability was not influenced by pH inhibition. It should be highlighted that in the previous test using the mesophilic dataset, the hydrogen inhibition term was included in the non-thermodynamic computation, but neither longer-chain acid accumulation nor process collapse could be observed.

### 3.2 Mechanisms for process collapse

As the expected signs for a manifest process collapse were absent consistently in the first set of results using the default mesophilic setup, a second series of testing was conducted. Here, possible mechanisms to provoke a process collapse in the simulation reactor corresponding to experimental observations were examined. Some aspects were identified that could lead to a process collapse through inhibition terms included in the ADM1, for example, by low pH values. However, based on experimental measurements, the conditions necessary for these inhibition terms with their respective constants to apply were not met; see for example, pH in the mesophilic reactor, which at its most extreme did not fall below 6.4 (Lebuhn et al., 2021). All computations included here considered the pH inhibition term as indicated in Table 1, which was insufficient to maintain and drive process instability toward process collapse. The same holds for ammonia inhibition and other inhibition terms included. Despite moderate pH values, the acid concentrations were high, leading to the supposition that direct inhibition by acid accumulation as included in $I_{\text{acids}}$ might be responsible for process collapse. Therefore, this second series of results compares the computation using the thermodynamic approach according to Equation 8 with two computations applying the inhibition term $I_{\text{acids}}$ to both methanogenic pathways (instead of only acetoclastic methanogenesis) and adapting the inhibition constant $K_{\text{acids}}$ .

**FIGURE 6:**
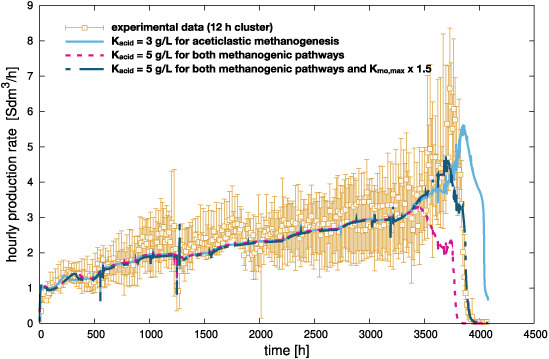
Comparison of methane production rates in standard cubic decimeters per hour for computations with the thermodynamic approach but a different setup of inhibition by total acids with parameter adjustment for $K_{\text{acids}}$ . Additionally, one computation with a 1.5-fold value for $K_{\text{mo},\text{ max}}$ in microbial growth inhibition is presented, yielding a relatively good timing of process collapse compared to experimental data.

First, a computation without the acid inhibition term was performed, showing that process instability via the thermodynamic considerations is still represented, although acid concentration peaks reached 60%, 70%, 64%, and 35% lower values for acetic, propionic, butyric, and valeric acid, respectively, compared to the computation with acid inhibition of the acetoclastic pathway. Applying the acid inhibition term for both methanogenic reactions with the initial constant $K_{\text{acids}}$ caused an early process instability and collapse between 500 h and 1000 h of simulated time (not shown), demonstrating that it is a possible mechanism for collapse. Accordingly, this mechanism was examined further, and the inhibition constant was varied to evaluate its impact on collapse timing. Results of these computations are presented in Figure 6 for the methane production rate and show clearly that applying inhibition by overall acid concentration to both methanogenic pathways with $K_{\text{acids}}=5$ g/L leads to a process collapse signified by a sudden fall in the methane production rate before feeding has stopped. For these computations, acid concentration profiles (not shown) increase during process instability and remain at high values after feeding has stopped, indicating a lasting process collapse. Choosing higher values for $K_{\text{acids}}$ caused further shifts in the time of process collapse, although the increase in methane production rate was not portrayed by the computational results, and corresponding values of $K_{\text{acids}}=8$ g/L are rather high.

Analysis of additional model output suggested that the limitation for microbial population density as included via $I_{\text{mo}}$ might be too strict, and additional computations were carried out. Included in the comparison of Figure 6 is an additional computation that adjusted $K_{\text{mo},\text{ max}}$ to produce a better fit for the timing of the observed process collapse. By using a 1.5-fold larger value for $K_{\text{mo},\text{ max}}$ , the microbial growth inhibition caused by high population densities takes effect at higher microbial concentrations in the reactor than with the previous value, allowing more microbial growth, including during overfeeding. It must be pointed out that these values were chosen by qualitative evaluation and might be better determined using a parameter fitting routine and validated experimentally in future work. Taking additional mechanisms into account will presumably require adjustments to ADM1 default values, which, except for maize silage-specific values for disintegration and hydrolysis, were used in this work without further adaptation. The concentration profiles of the third computation with a 1.5-fold larger value for $K_{\text{mo},\text{ max}}$ reflect what was observed in the methane production, already with a later onset of process collapse coinciding with the experimental values, although still overestimating final concentrations of most acids considered.

### 3.3 Parameter sensitivity analysis

**FIGURE 7:**
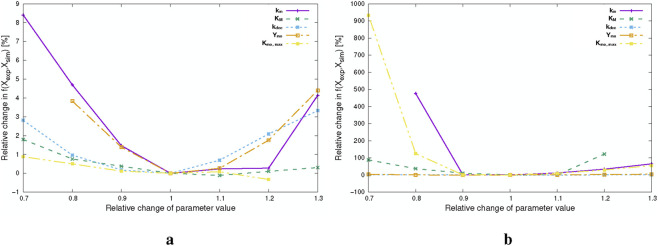
Influence of input parameters associated with methanogenesis on the model, quantified by the relative change of the target function $f\left(X_{\text{exp}},X_{\text{sim}}\right)$ between experimental and simulation results, evaluated over the entire runtime, including both stable and unstable reactor phases. **(a)** Acetoclastic methanogenesis. **(b)** Hydrogenotrophic methanogenesis.

Results for the local sensitivity analysis are presented as relative change of the target function $f\left(X_{\text{exp}},X_{\text{sim}}\right)$ plotted over the relative change of the respective input parameters in Figure 7a for parameters of acetoclastic and in Figure 7b for parameters of hydrogenotrophic methanogenesis. The results are based on the computation with the thermodynamic approach according to Jin and Bethke (2007), referenced in both series of results. The parameters are subjected to relative changes of ± 30%, which leads to a range of relative changes in the target function. Regarding acetoclastic methanogenesis, variation of input parameters exhibits most influence for the maximal uptake rate $k_{m}$ , the microbial growth fraction $Y_{\text{mo}}$ , and the respective decay rate $k_{\text{dec}}$ , with maximum differences amounting to less than 10%. Applying the same approach to the parameters of hydrogenotrophic methanogenesis indicates a much higher susceptibility of the model to these values, with some differences easily passing 100%. Prominent among the tested parameters are $K_{\text{mo},\text{ max}}$ , $k_{m}$ , and to a lesser degree, the Michaelis–Menten constant $K_{M}$ .

**FIGURE 8:**
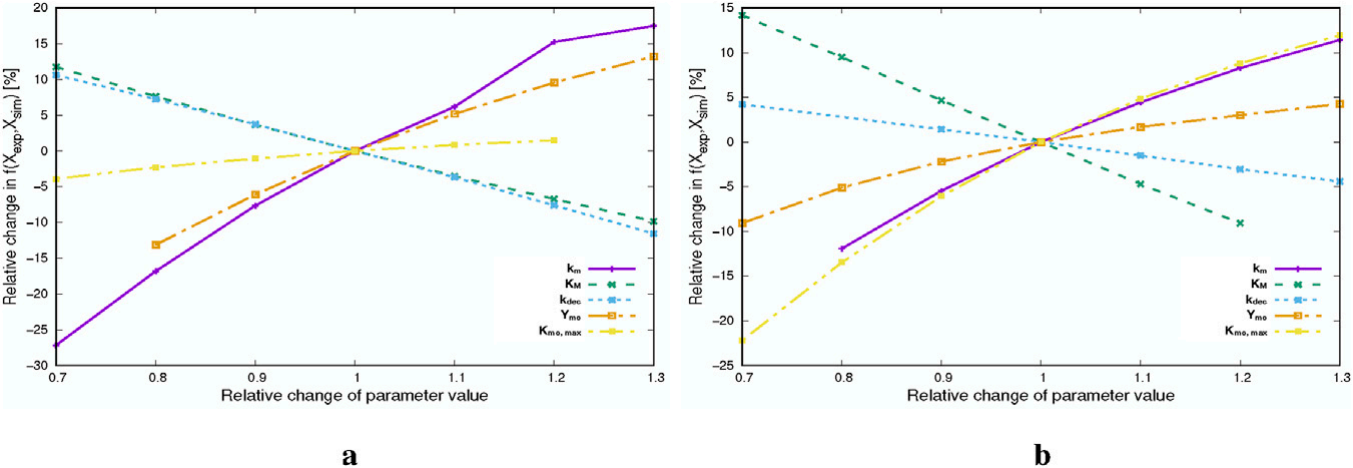
Influence of input parameters associated with methanogenesis on the model, evaluated only during the stable reactor phase up to 3000 h runtime. **(a)** Acetoclastic methanogenesis. **(b)** Hydrogenotrophic methanogenesis.

To further explore the impact of process instability in the model, the presented sensitivities are examined again with respect to the stable reactor phase only. To achieve this, the target function $f\left(X_{\text{exp}},X_{\text{sim}}\right)$ is evaluated only for data points before 3000 h runtime, predating process instability as indicated by acid accumulation. Figures 8a,b show that, with regard to only stable conditions, both methanogenic pathways display a similar impact on model sensitivity, both in terms of quantitative change and of qualitative parameter dependence. As negative values for the relative change indicate a better fit to experimental data for the chosen input parameter compared to the reference value, the stable phase sensitivity analysis presented in Figure 8 suggests adapting lower values for $k_{m}$ , $Y_{\text{mo}}$ , and $K_{\text{mo},\text{ max}}$ while increasing $K_{M}$ and $k_{\text{dec}}$ for both methanogenic reactions.

## 4 Discussion

### 4.1 Influence of thermodynamics

While the benefit of a thermodynamic approach to reaction rates does not become apparent in the simulated methane production rate, the acid concentration profiles highlight its necessity during process instability. Comparing the profiles for propionic, butyric, and valeric acid of the thermodynamic computations with those of the computation lacking a thermodynamic approach shows that the definitive accumulation of all considered acids can be observed only with a thermodynamic approach. Despite including hydrogen inhibition in the computation without thermodynamic rates, it shows no indication of process instability by an accumulation of longer-chain acids as their concentrations are far below the experimentally observed values, as pointed out by the black arrows in Figures 4b–d. As acetic acid accumulates in either case, the driving force for this accumulation is not mainly caused by thermodynamic effects. However, for the oxidation of longer-chain acids, the accumulation of acetate leads to thermodynamically unfavorable conditions and subsequently to acid build-up, suggesting the product surplus of acids as a primary inhibitory factor in the thermodynamic computations. Overall, the comparison of two computations with thermodynamic reaction rates, one computation without a thermodynamic approach, and experimental results point to the relevance of thermodynamic considerations in unstable reactor environments. Especially the accumulation of longer-chain acids such as propionic acid, which gave strong indications of instability in the experimental dataset, could only be reflected by taking thermodynamic corrections of the reaction rates into account.

Complementing these results, the computations using the thermophilic setup confirm that, in some cases, the thermodynamic treatment of reaction rates is necessary to reproduce the experimentally observed acid build-up. This is demonstrated by the unintentional process instability where experimental data showed an increase in longer-chain acids that could only be reproduced by the thermodynamic simulation. However, these complementary computations also demonstrate that a model without an explicit thermodynamic approach can be sufficient to reproduce process instabilities and even process collapse under some conditions, with the restriction that product inhibition by hydrogen is considered. This inhibition term represents a simplified approach based on thermodynamic considerations without regarding product inhibition by other products, such as acetic or longer-chain acids. Based on these observations, it could be argued that the process instability of the mesophilic reactor and the unintentional instability observed in the thermophilic reactor were primarily caused by acid accumulation, while the final instability of the thermophilic reactor was caused by an increase in hydrogen concentration. Overall, the model tests based on experimental data from stable and unstable reactor phases show that during stable conditions the thermodynamic approach generally is not needed, while some emerging and manifesting process instabilities are only reproduced by a thermodynamic model.

Going into greater detail for the mesophilic dataset, the overall lower values of acetic acid than experimental data in the stable reactor phase suggest an increased reduction of acetic acid compared to experimental results. On the other hand, lower acid concentrations in the computations do not result in above-average methane production, and the increase between 1000 h and 1500 h in the experimental data for acetic acid corresponds to the addition of digestate; see area A of Figure 3. It is therefore plausible to suppose a connection between the addition of digestate in the experimental reactor, the increased experimental values of acetic acid, and the increased methane production rate not replicated by the computations. Approaching instability, the slower increase of acid concentrations is in accordance with the slower increase and decrease in the methane production rate (see areas B and C in Figure 3), suggesting that the reduced amount of methane compared to experimental data and the computation without a thermodynamic approach is caused by a slower degradation of acetic acid. Of the two tested thermodynamic approaches, the computation using modified reaction rates based on Jin and Bethke (2007) provides a better fit to the experimental data, especially for propionic and valeric acid, with both computations overestimating the concentration of butyric acid considerably. This may be due to the fact that preceding acid-producing reactions are not treated thermodynamically, leading to acid production regardless of product accumulation, and the high carbohydrate content of maize silage leads to a high production of butyric acid via monosaccharides compared to valeric acid in the underlying reaction model. All three computations of the first series have in common that even significantly unstable reactor states recover after feeding has been suspended. This is apparent by the time delay between the experimentally observed collapse of methane production and the simulated results, and the decrease of accumulated acids in the computations. Both effects point to process recovery after feeding has stopped instead of a lasting process collapse.

### 4.2 Mechanisms for process collapse

While the model contains several possible mechanisms that could lead to a process collapse, these were not sufficient for the considered mesophilic problem setup. The second series of computations suggests that acid-dependent inhibition of both methanogenic pathways could be the responsible mechanism in the given dataset. In general, high acid concentrations will lead to low pH values, triggering pH-dependent inhibition in the existing model. However, with buffer substances, such as carbonate, the effect of accumulating acids manifests with a temporal delay, which explains the occurrence of moderate pH values in a reactor with high acid concentrations. The second series of results demonstrated that the moment of process collapse in the experimental data can be reflected by the computations by carrying out slight parameter adjustments. This encourages the utilization of parameter fitting routines in the future, for which some preparatory investigation is included in this work. Overall, the mechanism of methanogen inhibition by total acid concentrations provides an opportunity to qualitatively reproduce final process collapse in the considered computations based on experimental data.

### 4.3 Parameter sensitivity analysis

Results of the local sensitivity analysis for both methanogenic pathways show a higher impact of parameters belonging to hydrogenotrophic methanogenesis on the target function, with lower values of $K_{\text{mo},\text{ max}}$ and $k_{m}$ being prominent examples. Here, lower input values curb overall hydrogen turnover, and as hydrogenotrophic methanogenesis plays a key role in removing hydrogen from the digestate, which would otherwise inhibit acid oxidation processes, a manipulation of associated parameters necessarily influences acid concentrations. The large relative change in the target function for these parameter variations can be ascribed to the significant contribution of acid concentration values during the unstable reactor phase to $f\left(X_{\text{exp}},X_{\text{sim}}\right)$ , which results from their large absolute values. Comparing acid concentration profiles, both display a similar qualitative behavior with low amounts of acid present during the stable and relatively sudden increase during the unstable reactor phase, although with differently pronounced maxima (not shown). This difference in acid concentrations during process instability eclipses other variations during stable phases where absolute concentrations are lower. Judging from parameter sensitivities with regard to the entire simulation runtime, the given default parameters mostly have nearly optimal values as determined by finding the target function’s minimum. However, restricting sensitivity analysis to the stable reactor phase yields a very different picture. While relative changes of the target function are smaller overall, their behavior in dependence on parameter variations suggests minimal values outside the considered parameter range. This means that the given default parameters deviate from optimal values, that is, from values better reproducing acid concentrations during the stable reactor phase. Adapting input parameters would cause a slower degradation of acetic acid, leading to higher acid concentrations, which would better reflect experimental data. Consulting the more comprehensive sensitivity analysis, including stable and unstable process conditions, these parameter adjustments are not advisable as they will impair overall model performance. Especially, some of the hydrogenotrophic input parameters significantly affect model results where reactor instability comes into play and clearly impact acid concentrations. By implication, this means that optimal input parameters determined using experimental data for a stable reactor may not be able to predict varying reactor conditions accurately, or more precisely, that reproducing stable reactor behavior in a model could lead to substantial errors when approaching process instability. This must be kept in mind when parameter fitting routines are employed based on datasets describing different process conditions.

## 5 Conclusion

The investigation of the thermodynamic modeling approach on two datasets, each including stable and unstable process conditions, has demonstrated cases where accumulation of long-chain fatty acid could only be represented by including a thermodynamic correction. In the investigated mesophilic data, simulation results for propionic acid during process instability reached 111.04% of the experimental value with thermodynamic correction, while the uncorrected simulation represented less than 1%. A similar observation was made for the thermophilic dataset where an intermediate process instability occurred. The computation with the thermodynamic approach showed 82.91% of experimental propionic acid at a representative data point, while the computation without the thermodynamic correction showed only approximately 1%. Generally, including the thermodynamic modulation led to an accumulation of acids, especially longer-chain acids, providing clear indications of instability. Despite some differences from absolute experimental values, these findings highlight the importance of the thermodynamic approach in unstable reactor conditions when thermodynamically unfavorable product accumulations inhibit the reactions producing them.

The second series of results demonstrated that although the default values used in the investigation of the thermodynamic modeling approach did not produce a lasting process collapse, this can be achieved by considering inhibition by total acid concentrations for both methanogenic pathways. With the additional parameter adjustment for microbial growth inhibition, the methane production rate, including the sharp drop before feeding was terminated, compared well with the experimental data. The same holds for the acid concentration profiles, so that methanogen inhibition by total acid concentrations may be considered as the causal mechanism for process collapse in the presented case.

Compared to previous studies, this work included a thermodynamic correction to ADM1 reaction rates for all acid oxidation reactions and acetoclastic and hydrogenotrophic methanogenesis. Using two experimental examples with reactor instabilities, this work demonstrates that the thermodynamic correction can correctly predict long-chain fatty acid accumulation where the previous model failed. However, it also shows that this is not the case for all process instabilities as the thermophilic process collapse could be simulated as long as hydrogen inhibition, a simplified approach to the general thermodynamic consideration, was active and caused low pH values. Acid inhibition of both methanogenic pathways was shown as a possible mechanism for process collapse where pH inhibition is not dominant due to relatively stable pH while high acid concentrations are present.

## Data Availability

The data analyzed in this study are subject to the following licenses/restrictions: The data were generated during the Modisto project and are presented in Lebuhn et al. (2021). Some additional data were provided by the LfL scientists upon request. Requests to access these datasets should be directed to the project leader of Modisto, Michael Lebuhn, at michael.lebuhn@lfl.bayern.de.

## References

[B1] Adler P. Billig E. Brosowski A. Daniel-Gromke J. Falke I. Fischer E. (2014). Leitfaden Biogasaufbereitung und -einspeisung (Rostock: fachagentur Nachwachsende Rohstoffe e.V.), 5 edn.

[B2] Angelidaki I. Ellegaard L. Ahring B. K. (1999). A comprehensive model of anaerobic bioconversion of complex substrates to biogas. Biotechnol. Bioeng. 63, 363–372. 10.1002/(sici)1097-0290(19990505)63:3<363::aid-bit13>3.0.co;2-z 10099616

[B3] Batstone D. Keller J. Angelidaki I. Kalyuzhnyi S. V. Pavlostathis S. G. Rozzi A. (2002). The IWA anaerobic digestion model No 1 (ADM1). Water Sci. Technol. 45, 65–73. 10.2166/wst.2002.0292 12188579

[B4] Batstone D. Keller J. Steyer J.-P. (2006). A review of ADM1 extensions, applications, and analysis: 2002-2005. Water Science Technology A Journal Int. Assoc. Water Pollut. Res. 54, 1–10. 10.2166/wst.2006.520 17037164

[B5] Gehring T. Niedermayr A. Berzio S. Immenhauser A. Wichern M. Lübken M. (2016). Determination of the fractions of syntrophically oxidized acetate in a mesophilic methanogenic reactor through an 12C and 13C isotope-based kinetic model. Water Res. 102, 362–373. 10.1016/j.watres.2016.06.038 27390036

[B6] Hackbusch W. (1985). Multi-Grid Methods and Applications. 1 edn. Berlin Heidelberg: Springer.

[B7] Hanselmann K. (1991). Microbial energetics applied to waste repositories. Experientia 47, 645–687. 10.1007/bf01958816

[B8] Hoh C. Cord-Ruwisch R. (1996). A practical kinetic model that considers endproduct inhibition in anaerobic digestion processes by including the equilibrium constant. Biotechnol. Bioeng. 51, 597–604. 10.1002/(SICI)1097-0290(19960905)51:5<597::AID-BIT12>3.0.CO;2-F 18629824

[B9] Jeppsson U. Rosen C. (2006). Aspects on ADM1 Implementation Within the BSM2 Framework.

[B10] Jin Q. Bethke C. (2007). The thermodynamics and kinetics of microbial metabolism. Am. J. Sci. 307, 643–677. 10.2475/04.2007.01

[B11] Kelif Ibro M. Ramayya Ancha V. Beyene Lemma D. (2024). Biogas production optimization in the anaerobic codigestion process: a critical review on process parameters modeling and simulation tools. J. Chem. 2024, 4599371–25. 10.1155/2024/4599371

[B12] Koch K. Lübken M. Gehring T. Wichern M. Horn H. (2010). Biogas from grass silage – measurements and modeling with ADM1. Bioresour. Technol. 101, 8158–8165. 10.1016/j.biortech.2010.06.009 20580224

[B13] Landolt H. Börnstein R. (1971). “Zahlenwerte und Funktionen aus Physik, Chemie, Astronomie, Geophysik, Technik,” 2/4. Berlin Göttingen Heidelberg: Springer.

[B14] Lebuhn M. Munk B. Rettenmaier R. Horn M. Klocke B. Zinser C. (2021). Schlussbericht Zum Verbundvorhaben, Thema: Dynamik Des Intermediat-Stoffwechsels in Biogasprozessen (MODISTO). Fachagentur Nachwachsende Rohst. e.V. (FNR). Available online at: https://biogas.fnr.de/index.php?id=11390&fkz=22003513.

[B15] Lechner M. (1992). Taschenbuch Für Chemiker Und Physiker (Berlin Heidelberg: Springer), 4 edn.

[B16] Muha I. (2012). Modellierung Und Simulation Eines Biogasreaktors. Johann Wolfgang Goethe-Universität Frankfurt am Main. Dissertation.

[B17] Muha I. Grillo A. Heisig M. Schönberg M. Linke B. Wittum G. (2012). Mathematical modeling of process liquid flow and acetoclastic methanogenesis under mesophilic conditions in a two-phase biogas reactor. Bioresour. Technol. 106, 1–9. 10.1016/j.biortech.2011.11.087 22206918

[B18] Munk B. (2019). Raw data of measurements and laboratory analyses. Private communication and data sharing within the modisto project, processed data published in final report of modisto

[B19] Reiter S. Vogel A. Heppner I. Rupp M. Wittum G. (2014). A massively parallel geometric multigrid solver on hierarchically distributed grids. Comput. Vis. Sci. 16, 151–164. 10.1007/s00791-014-0231-x

[B20] Rosen C. Vrecko D. Gernaey K. V. Pons M. N. Jeppsson U. (2006). Implementing ADM1 for plant-wide benchmark simulations in matlab/simulink. Water Science Technology A Journal Int. Assoc. Water Pollut. Res. 54, 11–19. 10.2166/wst.2006.521 17037165

[B21] Rüppel N. (2019). Modisto operating log. Private communication and data sharing within the modisto project, processed data published in final report of modisto

[B22] Schink B. (1997). Energetics of syntrophic cooperation in methanogenic degradation. Microbiol. Mol. Biol. Rev. 61, 262–280. 10.1128/mmbr.61.2.262-280.1997 9184013 PMC232610

[B23] Siegrist H. Vogt D. Garcia-Heras J. L. Gujer W. (2002). Mathematical model for meso- and thermophilic anaerobic sewage sludge digestion. Environ. Sci. and Technol. 36, 1113–1123. 10.1021/es010139p 11917999

[B24] Thauer R. Jungermann K. Decker K. (1977). Energy conservation in chemotrophic anaerobic bacteria. Bacteriol. Rev. 41, 100–180. 10.1128/br.41.1.100-180.1977 860983 PMC413997

[B25] Vogel A. (2014). Flexible Und Kombinierbare Implementierung Von Finite-Volumen-Verfahren Höherer Ordnung. Johann Wolfgang Goethe-Universität. Frankfurt am Main. Dissertation.

[B26] Vogel A. Reiter S. Rupp M. Nägel A. Wittum G. (2014). UG 4: a novel flexible software system for simulating PDE based models on high performance computers. Comput. Vis. Sci. 16, 165–179. 10.1007/s00791-014-0232-9

[B27] Wittum R. (2025). Modellierung Anaerober Abbauprozesse in Biogasanlagen Unter Berücksichtigung Thermodynamischer Begrenzungen in Räumlich Aufgelösten Reaktoren. Johann Wolfgang Goethe-Universität Frankfurt am Main. Dissertation.

