# Supplementary material for: Modeling process instability in anaerobic digestion by using a thermodynamically corrected model
Source: Front Bioeng Biotechnol. 2026 Aug 4;14:1880446. doi: 10.3389/fbioe.2026.1880446 (PMC13481626; doi:10.3389/fbioe.2026.1880446)
Supplement: Supplementary file 1 [file DataSheet1.pdf]

Supplementary Material

1 CHOICE OF STOICHIOMETRIC PARAMETERS

The stoichiometry of substrate disintegration depends on its composition and is expressed by mass-based stoichiometric coefficients μ_i^j for each product i of the primary reactant j. In the presented model any substrate is assumed to be composed of three degradable material groups: carbohydrates, lipids, and proteins, and an inert fraction which cannot be broken down further. Extended Weender analysis yields values to describe raw protein (RP) and raw lipid (RL) content of a given substrate so that the corresponding stoichiometric factors may be derived dependent on its volatile solid content (VS) as

$$\mu_{\text{proteins}}^{\text{substrate}} = \frac{\text{RP}}{\text{VS}} \tag{S1}$$

$$\mu_{\text{lipids}}^{\text{substrate}} = \frac{\text{RL}}{\text{VS}} \tag{S2}$$

according to Koch et al. (2010). For carbohydrates starch content $\text{RF} + \text{NfE} - \text{NDF}$ as well as degradation level d of summed up cellulose and hemicellulose content $\text{NDF} - \text{ADL}$ are considered, leading to

$$\mu_{\text{carbohydrates}}^{\text{substrate}} = \frac{(\text{RF} + \text{NfE} - \text{NDF}) + (\text{NDF} - \text{ADL}) \cdot d}{\text{VS}}. \tag{S3}$$

In order to satisfy conservation of mass the stoichiometric coefficient for any substrates inert fraction is given by

$$\mu_{\text{inert}}^{\text{substrate}} = 1 - \mu_{\text{carbohydrates}} - \mu_{\text{proteins}} - \mu_{\text{lipids}}. \tag{S4}$$

Analyses of the maize silage used as substrate in the experimental reactors provided the necessary data for determining disintegration stoichiometry via the procedure outlined here, yielding

$$\mu_{\text{proteins}}^{\text{substrate}} = 0.083$$

$$\mu_{\text{lipids}}^{\text{substrate}} = 0.035$$

$$\mu_{\text{carbohydrates}}^{\text{substrate}} = 0.746$$

with respect to volatile content of the substrate and assuming a degradation level $d = 0.864$. For hydrolytic reactions the mass-based stoichiometries of carbohydrate and protein degradations with a single reactant and single product each are simply $\mu_{\text{monosaccharides}}^{\text{carbohydrates}} = 1$ and $\mu_{\text{aminoacids}}^{\text{proteins}} = 1$ respectively while the hydrolysis of lipids presents a more interesting case with the involvement of multiple products. The ADM1 suggests that $95\,\%$ of the lipids, measured in chemical oxygen demand, are reduced to long chain fatty acids (LCFA) while the remaining $5\,\%$ are reduced to monosaccharides. Unit transformation from kgCOD product per kgCOD lipids to gram product per gram lipids yields $\mu_{\text{lcfa}}^{\text{lipids}} = 0.895$ and $\mu_{\text{monosaccharides}}^{\text{lipids}} = 0.105$. Taking palmitic acid as a representative substance for long chain fatty acids its production from tripalmitin is described by the reaction

$$C_{51}H_{98}O_6 + 3\,H_2O \longrightarrow 3\,C_{16}H_{32}O_2 + \frac{1}{2}\,C_6H_{12}O_6 + H_2, \tag{S5}$$

yielding three moles palmitic acid, half a mole glucose as well as one mole hydrogen. Based on this chemical equation the stoichiometry of lipid hydrolysis in this work includes the produced mass of hydrogen as well. After unit transformation from mol to grams this yields $\mu^{\text{lipids}}_{\text{lcfa}} = 0.893$, $\mu^{\text{lipids}}_{\text{monosaccharides}} = 0.105$ and $\mu^{\text{lipids}}_{\text{hydrogen}} = 0.002$, not too dissimilar to the ADM1 approach.

Model stoichiometry for acidogenesis of monosaccharides is derived analogously to the ADM1 procedure by merging three known chemical reactions of glucose with different weights into one equation. In reference to a single mole of glucose it takes the following form

$$\begin{aligned} C_6H_{12}O_6 + 0.977\,H_2O \longrightarrow\ & 0.156\,CH_3(CH_2)_2COOH + 0.462\,CH_3CH_2COOH \\ & + 1.227\,CH_3COOH + 1.538\,CO_2 \\ & + 2.304\,H_2 + 0.231\,H_2O. \end{aligned} \tag{S6}$$

Similarly the stoichiometry for acidogenesis of amino acids is a conglomeration of multiple stoichiometric coefficients based on protein composition of standard amino acids according to (Ramsay, 1997, tab. 3.2). As aromatic substances are not considered in the model their fraction is transferred to acetic acid based on carbon content. This yields the effective chemical equation

$$\begin{aligned} \text{amino acids} \longrightarrow\ & 0.213\,CH_3(CH_2)_3COOH + 0.259\,CH_3(CH_2)_2COOH \\ & 0.096\,CH_3CH_2COOH + 0.979\,CH_3COOH \\ & + 0.819\,CO_2 + 0.660\,H_2 \end{aligned} \tag{S7}$$

per mol amino acids, which are estimated to have a molar mass of 133 g/mol using the same information on protein composition in (Ramsay, 1997, tab. 3.2). As long chain fatty acids (LCFA) are represented by palmitic acid, the reaction

$$C_{16}H_{32}O_2 + 14\,H_2O \longrightarrow 8\,CH_3COOH + 14\,H_2 \tag{S8}$$

serves as basis to determine stoichiometric coefficients for LCFA acidogenesis. Stoichiometry of acetogenic and methanogenic processes are based on the following chemical reactions without additional revisions:

oxidation of valeric acid:

$$CH_3(CH_2)_3COOH + 2\,H_2O \longrightarrow CH_3CH_2COOH + CH_3COOH + 2\,H_2 \tag{S9}$$

oxidation of butyric acid:

$$CH_3(CH_2)_2COOH + 2\,H_2O \longrightarrow 2\,CH_3COOH + 2\,H_2 \tag{S10}$$

oxidation of propionic acid:

$$CH_3CH_2COOH + 2\,H_2O \longrightarrow CH_3COOH + CO_2 + 3\,H_2 \tag{S11}$$

aceticlastic methanogenesis:

$$CH_3COOH \longrightarrow CH_4 + CO_2 \tag{S12}$$

oxidation of acetic acid:

$$CH_3COOH + 2\,H_2O \longrightarrow 2\,CO_2 + 4\,H_2 \tag{S13}$$

hydrogenotrophic methanogenesis:

$$CO_2 + 4\,H_2 \longrightarrow CH_4 + 2\,H_2O. \tag{S14}$$

2 TRANSFORMATION OF KINETIC PARAMETERS

Kinetic parameters correspond to ADM1 values presented by Rosen et al. (2006); Jeppsson and Rosen (2006) but have been adapted to suit the unit requirements by transforming parameters based on chemical oxygen demand. This is achieved by considering molar mass and theoretical oxygen demand for model substances which can be estimated for a substance $\mathrm{C}_a\mathrm{H}_b\mathrm{O}_c\mathrm{N}_d$ according to Koch et al. (2010) by

$$\mathrm{ThOD} = \frac{16(2a + 0.5(b - 3d) - c)}{12a + b + 16c + 14d} \left[\frac{\mathrm{g}_{\mathrm{O}_2}}{\mathrm{g}_{\mathrm{C}_a\mathrm{H}_b\mathrm{O}_c\mathrm{N}_d}}\right] \tag{S15}$$

thus allowing a transformation from units of kgCOD into g. With molar masses and above equation for ThOD the transformed kinetic parameters included in tables S1 were computed from ADM1 values. Transformation of ADM1 inhibition constants yield $K_{\mathrm{IN}} = 0.001805$ g/L for all catalyzed reactions, $K_{\mathrm{NH}_3} = 33.07 \cdot 10^{-2}$ g/L for aceticlastic methanogenesis, $K_{\mathrm{H}_2} = 6.25 \cdot 10^{-7}$ g/L for the acidogenesic of LCFA, $K_{\mathrm{H}_2} = 4.38 \cdot 10^{-7}$ g/L for the oxidation of propionic acid and $K_{\mathrm{H}_2} = 1.25 \cdot 10^{-6}$ g/L for all other oxidation reactions. In computations with a thermodynamic approach no additional hydrogen inhibition is considered in acido- and acetogenesis reactions. Based on Weinrich (2017), a default value of $K_{\mathrm{acids}} = 3$ g/L for aceticlastic methanogenesis is chosen and, adapted from Muha et al. (2012), $K_{\mathrm{mo,\,max}} = 10.5$ g/L is set as default for all enzymatic reactions. For the computations deviating from these defaults the respective approach and values are stated explicitly in the text.

3 ADDITIONAL PHYSICOCHEMICAL CONSTANTS

In addition to quantities such as molar mass or Gibbs free energy, values for the acid dissociation constants, Henry coefficients of gases as well as diffusion coefficients for gas phase and liquid model substances are required. Most of these values are readily available and in part already included in the ADM1 such as acid dissocation constants and the self-ionization constant for water and therefore not repeated here (Batstone et al., 2002). The computed values for the Henry coefficients are based on tabulated values by Sander (2015) and transformed into coefficients describing the unit-less concentration fraction H^{cc} depending on a given temperature and at atmospheric pressure. Diffusion coefficients vary between substances and for some applications in modeling their differences are essential to the examined processes. In the presented work with focus on the thermodynamic aspects, no density-driven flow and the assumption of an homogenized digestate the intricacies of substance transport were not particularly emphasized. As a simplification, which might not be applicable to a different model setup, all substances in the liquid phase were assigned the same diffusion coefficient $\mathbb{D} = 4.356 \cdot 10^{-4}\,\mathrm{dm}^2/\mathrm{h}$ as a default value as suggested for acetic acid by Muha et al. (2012). While the process of digestate mixing was not considered via a convection term as the time scale is very small compared to reaction kinetics it could be represented by accelerated diffusion, i.e. a larger diffusion coefficient. Preliminary testing of this hypothesis for the presented model problem did not show significant deviations, so for liquid substances the coefficient stated before was used. For dissolved hydrogen, carbon dioxide and methane the diffusion coefficients in water, calculated using the Wilke-Chang relation (Wang et al., 2007), move in the range of 10^{-4} to $10^{-3}\,\mathrm{dm}^2/\mathrm{h}$, though here results are impacted by digestate mixing, accelerated transport and especially transfer to reactor headspace. Empirically a value approximately two orders of magnitude faster than the coefficient of pure diffusion has been roughly determined so that the presented computations were performed using $\mathbb{D}_i^l = 2.77 \cdot 10^{-1}\,\mathrm{dm}^2/\mathrm{h}$. A possible phase change of dissolved gases into their free form while in the digestate with subsequent transport into the headspace might explain this order of magnitude between diffusion in water and self diffusion of gases. The

**first order kinetics**	k^r $\left[\frac{1}{\mathrm{h}}\right]$			
disintegration of substrate	0.032			
hydrolysis of carbohydrates	0.029			
hydrolysis of proteins	0.010			
hydrolysis of lipids	0.005			
**enzyme kinetics**	k^r_{m} $\left[\frac{\mathrm{g_{reactant}}}{\mathrm{g_{mo}\,h}}\right]$	K^r_{M} $\left[\frac{\mathrm{g}}{\mathrm{L}}\right]$	Y_{mo} $\left[\frac{\mathrm{g_{mo}}}{\mathrm{g_{reactant}}}\right]$	k_{dec} $\left[10^{-4}\ \frac{1}{\mathrm{h}}\right]$
acidogenesis of monosaccharides	1.6006	0.469	0.0781	8.3
acidogenesis of amino acid	2.0097	0.212	0.0829	8.3
acidogenesis of LCFA	0.1179	0.138	0.1272	8.3
oxidation of valeric acid	0.5582	0.098	0.0896	8.3
oxidation of butyric acid	0.6260	0.110	0.0799	8.3
oxidation of propionic acid	0.4888	0.066	0.0443	8.3
aceticlastic methanogenesis	0.4268	0.141	0.0390	8.3
oxidation of acetic acid[a]	0.1067	0.141	0.0625	8.3
hydrogenotrophic methanogenesis	0.2490	$8.75\cdot10^{-7}$	0.3514	8.3

[a] values based on Gehring et al. (2016).

Table S1. Overview of transformed kinetic parameters used in the model application presented in this work. Excepting values for the oxidation of acetic acid, as noted in the table, all values of enzymatic reactions are based on ADM1 values from Batstone et al. (2002); Jeppsson and Rosen (2006). Parameters for first order equations are based on maize silage specific values taken from Biernacki et al. (2013).

latter ranges around 10^0 and $10^1\,\mathrm{dm}^2/\mathrm{h}$ for different gaseous substances, see for example Mostinsky (2011) or generally for diffusion in gases, Haynes (2014), and as a simplification in this work $\mathbb{D}^g_i = 5\,\mathrm{dm}^2/\mathrm{h}$ was chosen for all three model gases.

REFERENCES

Batstone, D., Keller, J., et al. (2002). The IWA Anaerobic Digestion Model No 1 (ADM1). *Water Science and Technology* 45, 65. doi:10.2166/wst.2002.0292

Biernacki, P., Steinigeweg, S., et al. (2013). Application of anaerobic digestion model no. 1 for describing anaerobic digestion of grass, maize, green weed silage, and industrial glycerine. *Bioresource Technology* 127, 188–194. doi:https://doi.org/10.1016/j.biortech.2012.09.128

Gehring, T., Niedermayr, A., et al. (2016). Determination of the fractions of syntrophically oxidized acetate in a mesophilic methanogenic reactor through an 12C and 13C isotope-based kinetic model. *Water Research* 102, 362 – 373. doi:https://doi.org/10.1016/j.watres.2016.06.038

Haynes, W. (2014). *CRC Handbook of Chemistry and Physics*. CRC Handbook of Chemistry and Physics (CRC Press)

Jeppsson, U. and Rosen, C. (2006). Aspects on ADM1 Implementation within the BSM2 Framework

Koch, K., Lübken, M., et al. (2010). Biogas from grass silage – Measurements and modeling with ADM1. *Bioresource Technology* 101, 8158 – 8165. doi:https://doi.org/10.1016/j.biortech.2010.06.009

Mostinsky, I. L. (2011). Diffusion coefficient. *A-to-Z guide to Thermodynamics, Heat & Mass Transfer, and Fluid Engineering* `https://www.thermopedia.com/content/696/`. Last accessed on 03/18/2024.

Muha, I., Grillo, A., et al. (2012). Mathematical modeling of process liquid flow and acetoclastic methanogenesis under mesophilic conditions in a two-phase biogas reactor. *Bioresource Technology* 106, 1–9. doi:https://doi.org/10.1016/j.biortech.2011.11.087

Ramsay, I. R. (1997). *Modelling and Control of High-Rate Anaerobic Wastewater Treatment Systems*. Dissertation, University of Queensland

Rosen, C., Vrecko, D., et al. (2006). Implementing ADM1 for plant-wide benchmark simulations in Matlab/Simulink. *Water science and technology : a journal of the International Association on Water Pollution Research* 54, 11–9. doi:10.2166/wst.2006.521

Sander, R. (2015). Compilation of Henry's law constants (version 4.0) for water as solvent. *Atmospheric Chemistry and Physics* 15, 4399–4981. doi:10.5194/acp-15-4399-2015

Wang, T.-F., Kasting, G. B., and Nitsche, J. M. (2007). A multiphase microscopic diffusion model for stratum corneum permeability. ii. estimation of physicochemical parameters, and application to a large permeability database. *Journal of Pharmaceutical Sciences* 96, 3024–3051. doi:https://doi.org/10.1002/jps.20883

Weinrich, S. (2017). *Praxisnahe Modellierung von Biogasanlagen - Systematische Vereinfachung des Anaerobic Digestion Model No. 1 (ADM1)*. Dissertation, Universität Rostock
