# Supplementary material for: Modeling process instability in anaerobic digestion by using a thermodynamically corrected model
Source: Front Bioeng Biotechnol. 2026 Aug 4;14:1880446. doi: 10.3389/fbioe.2026.1880446 (PMC13481626; doi:10.3389/fbioe.2026.1880446)
Supplement: Supplementary file 2 [file DataSheet10.pdf]

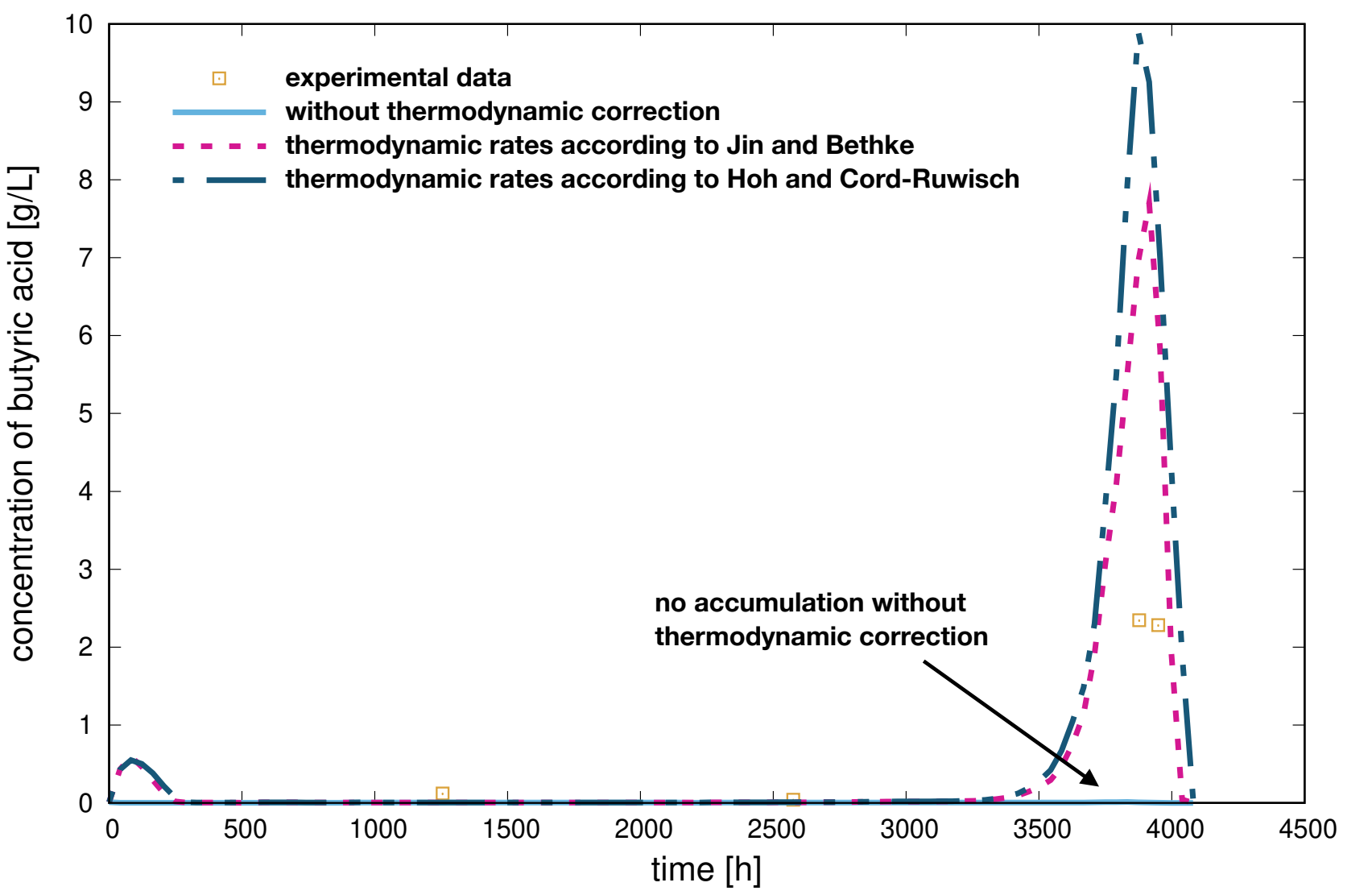

experimental data
without thermodynamic correction
thermodynamic rates according to Jin and Bethke
thermodynamic rates according to Hoh and Cord-Ruwisch
no accumulation without
thermodynamic correction
concentration of butyric acid [g/L]
time [h]
0
1
2
3
4
5
6
7
8
9
10
0
500
1000
1500
2000
2500
3000
3500
4000
4500
