# Supplementary material for: Modeling process instability in anaerobic digestion by using a thermodynamically corrected model
Source: Front Bioeng Biotechnol. 2026 Aug 4;14:1880446. doi: 10.3389/fbioe.2026.1880446 (PMC13481626; doi:10.3389/fbioe.2026.1880446)
Supplement: Supplementary file 5 [file DataSheet13.pdf]

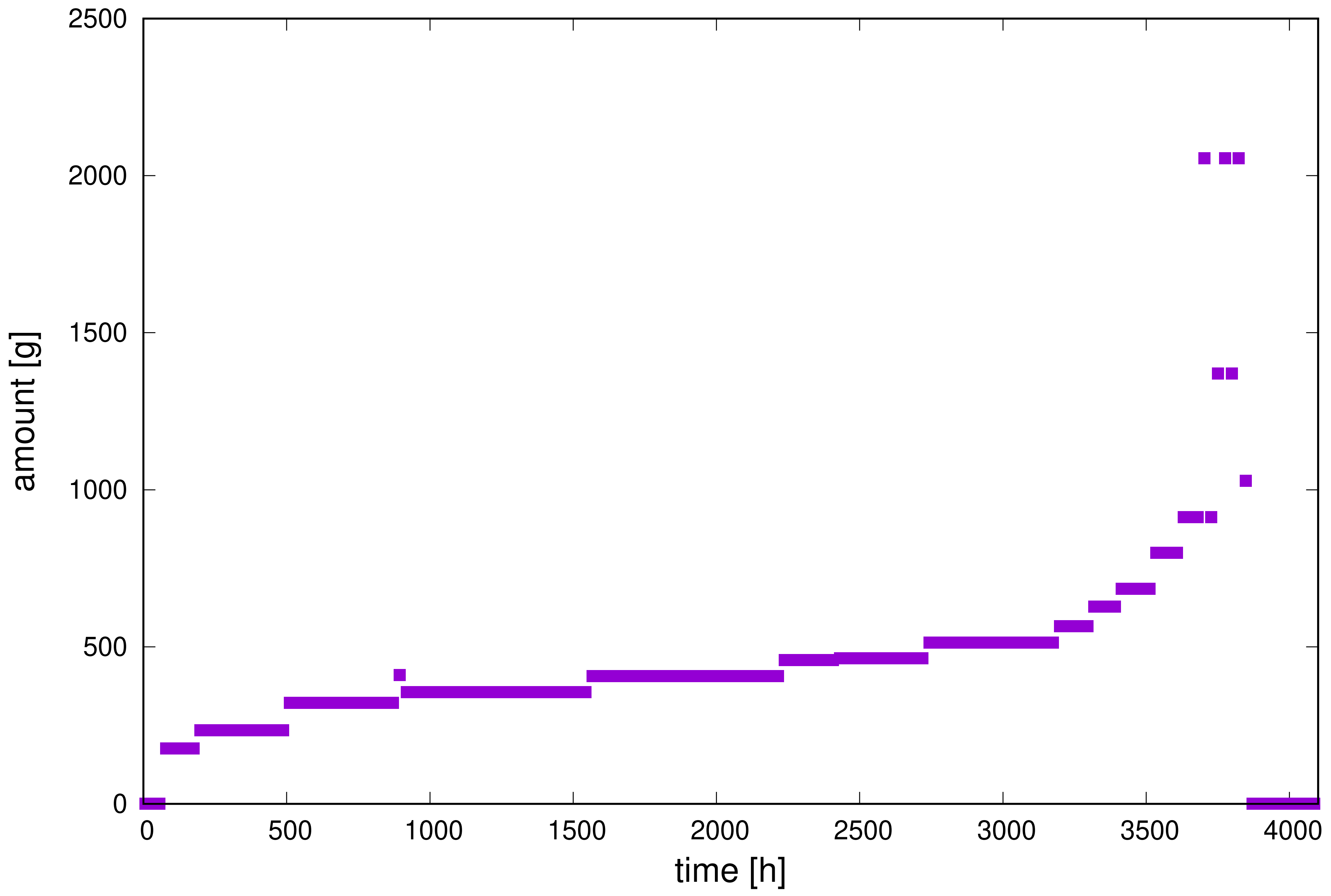

amount [g]
2500
2000
1500
1000
500
0
0
500
1000
1500
2000
2500
3000
3500
4000
time [h]
