# Supplementary material for: Modeling process instability in anaerobic digestion by using a thermodynamically corrected model
Source: Front Bioeng Biotechnol. 2026 Aug 4;14:1880446. doi: 10.3389/fbioe.2026.1880446 (PMC13481626; doi:10.3389/fbioe.2026.1880446)
Supplement: Supplementary file 6 [file DataSheet14.pdf]

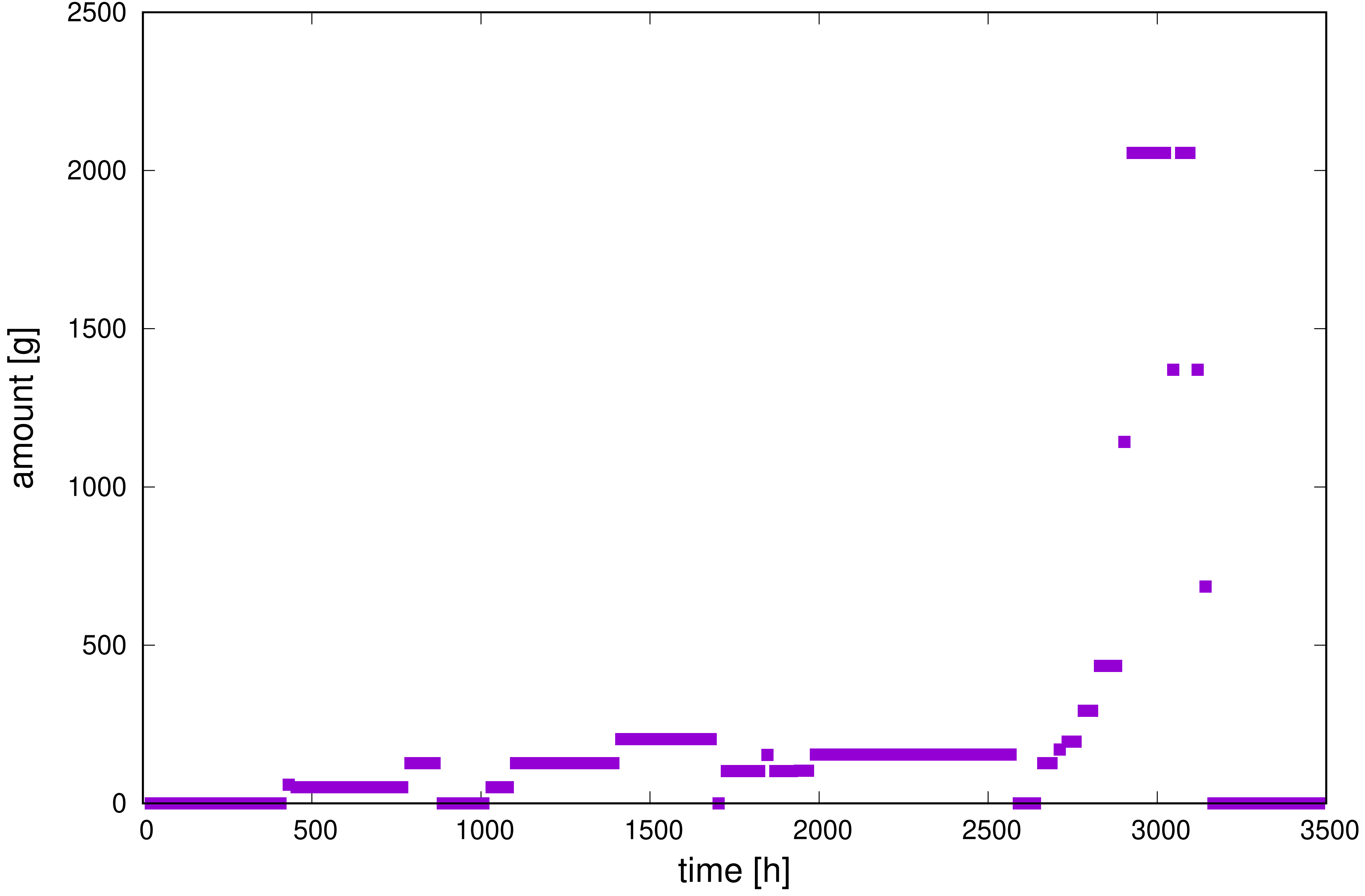

2500
2000
1500
1000
500
0
amount [g]
0
500
1000
1500
2000
2500
3000
3500
time [h]
