# Supplementary material for: Modeling process instability in anaerobic digestion by using a thermodynamically corrected model
Source: Front Bioeng Biotechnol. 2026 Aug 4;14:1880446. doi: 10.3389/fbioe.2026.1880446 (PMC13481626; doi:10.3389/fbioe.2026.1880446)
Supplement: Supplementary file 8 [file DataSheet3.pdf]

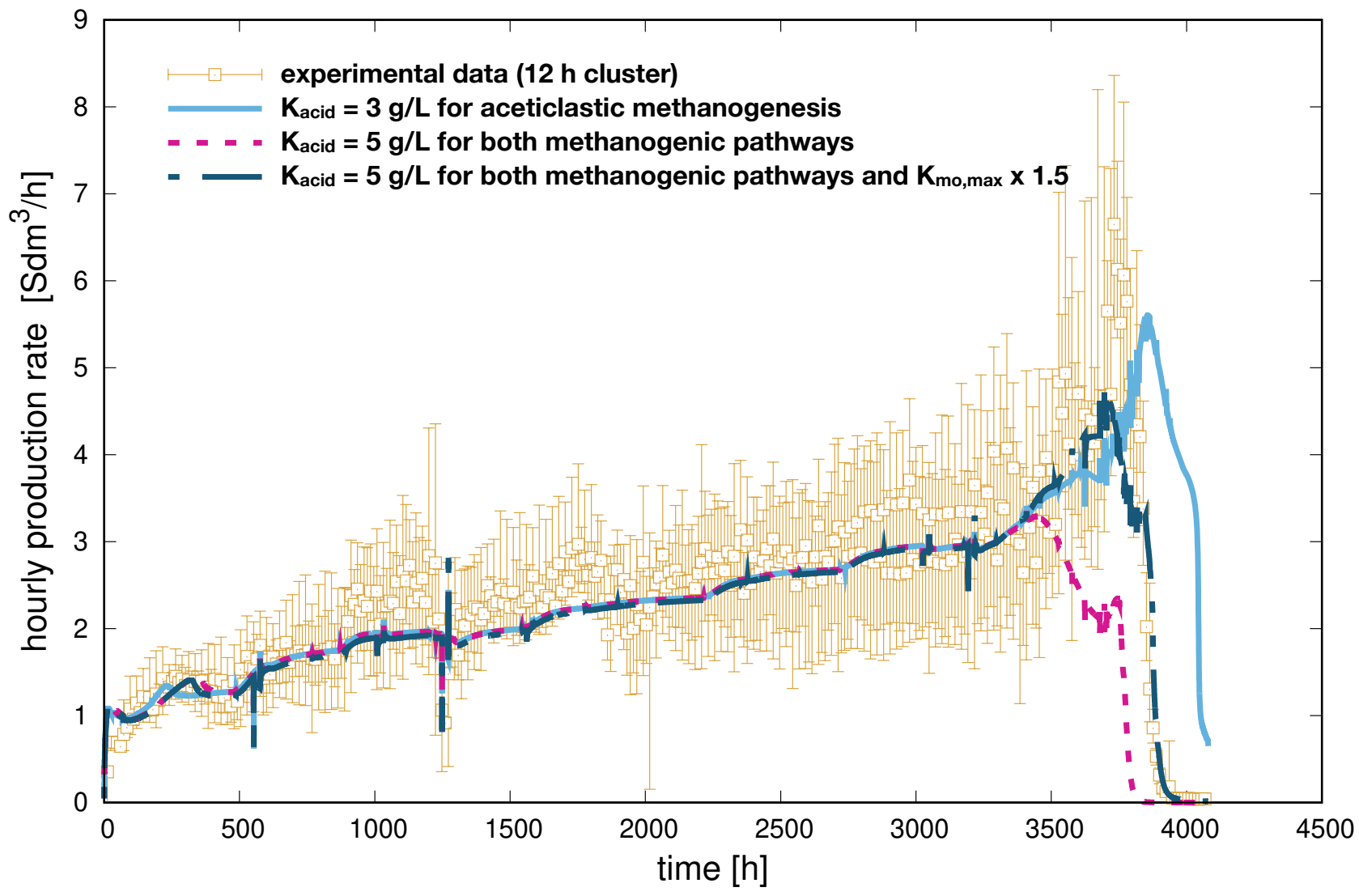
experimental data (12 h cluster)
K_{acid} = 3 g/L for aceticlastic methanogenesis
K_{acid} = 5 g/L for both methanogenic pathways
K_{acid} = 5 g/L for both methanogenic pathways and $K_{mo,max}$ x 1.5
hourly production rate [Sdm^3/h]
time [h]
0
1
2
3
4
5
6
7
8
9
0
500
1000
1500
2000
2500
3000
3500
4000
4500
