# Supplementary material for: Modeling process instability in anaerobic digestion by using a thermodynamically corrected model
Source: Front Bioeng Biotechnol. 2026 Aug 4;14:1880446. doi: 10.3389/fbioe.2026.1880446 (PMC13481626; doi:10.3389/fbioe.2026.1880446)
Supplement: Supplementary file 10 [file DataSheet5.pdf]

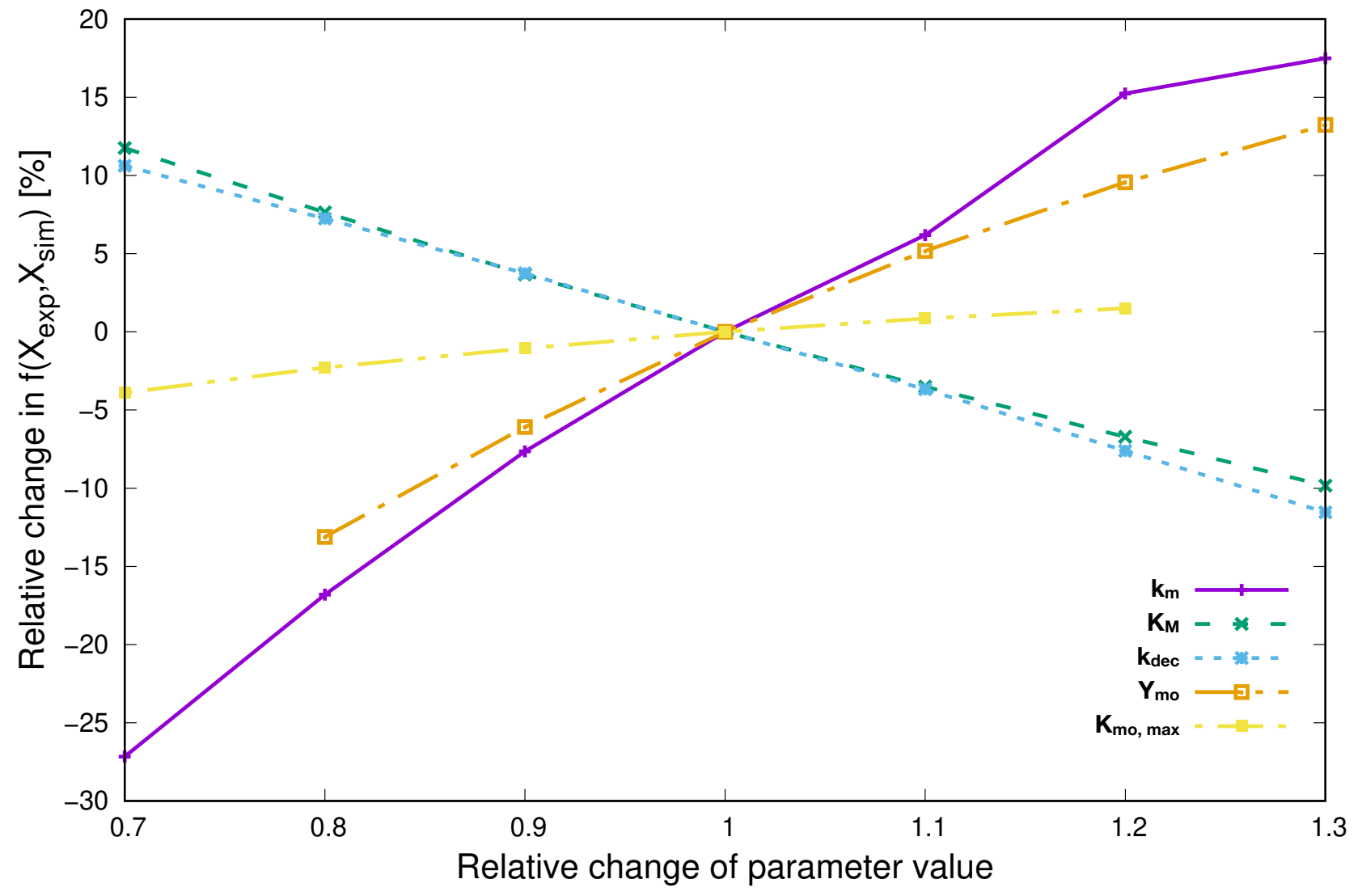

Relative change in f(Xexp,Xsim) [%]
20
15
10
5
0
−5
−10
−15
−20
−25
−30
0.7
0.8
0.9
1
1.1
1.2
1.3
Relative change of parameter value
km
KM
kdec
Ymo
Kmo, max
