# Supplementary material for: Modeling process instability in anaerobic digestion by using a thermodynamically corrected model
Source: Front Bioeng Biotechnol. 2026 Aug 4;14:1880446. doi: 10.3389/fbioe.2026.1880446 (PMC13481626; doi:10.3389/fbioe.2026.1880446)
Supplement: Supplementary file 11 [file DataSheet6.pdf]

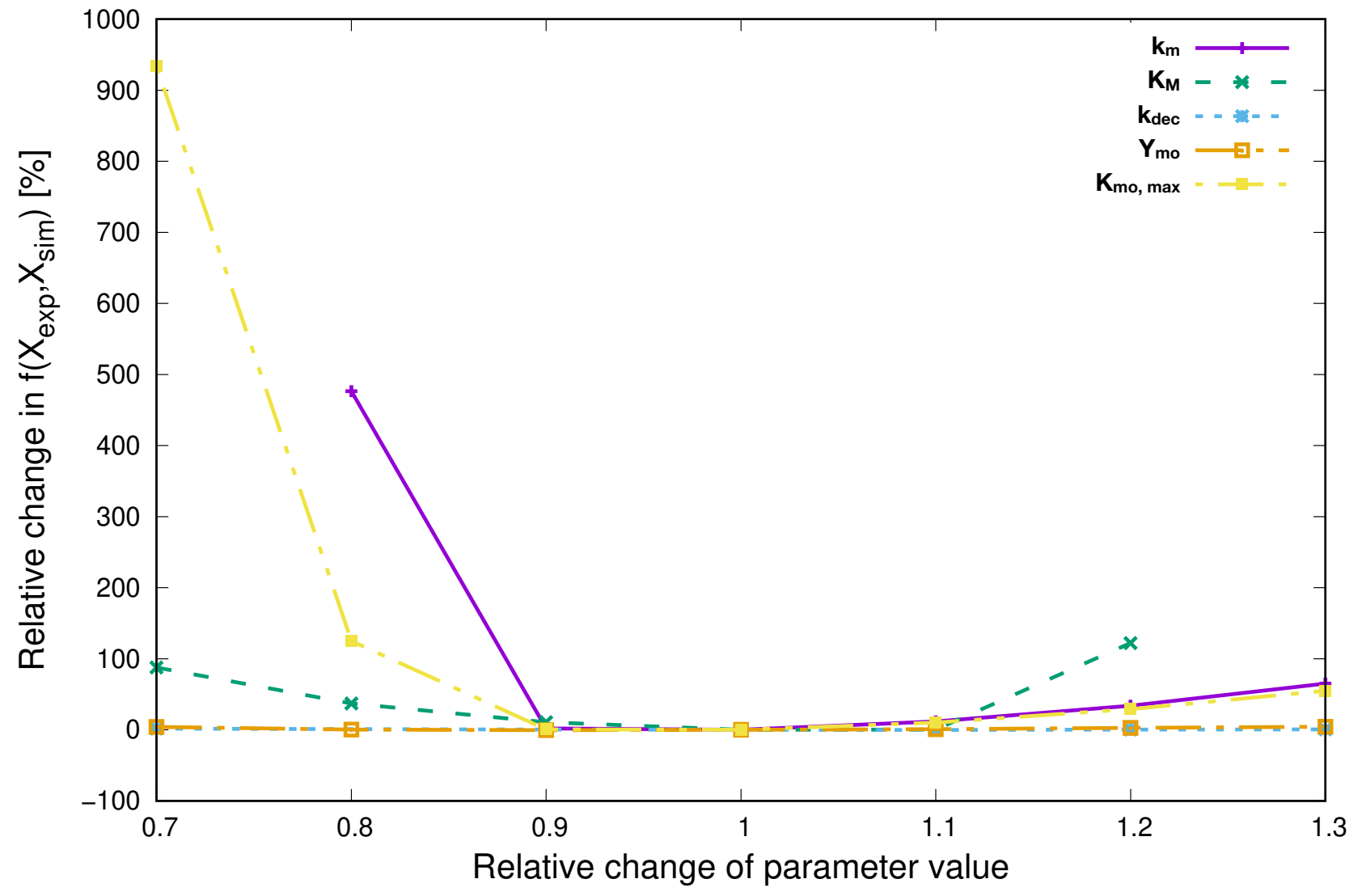

Relative change in f(X_exp,X_sim) [%]
Relative change of parameter value
k_m
K_M
k_dec
Y_mo
K_mo, max
1000
900
800
700
600
500
400
300
200
100
0
−100
0.7
0.8
0.9
1
1.1
1.2
1.3
