# Supplementary material for: Modeling process instability in anaerobic digestion by using a thermodynamically corrected model
Source: Front Bioeng Biotechnol. 2026 Aug 4;14:1880446. doi: 10.3389/fbioe.2026.1880446 (PMC13481626; doi:10.3389/fbioe.2026.1880446)
Supplement: Supplementary file 12 [file DataSheet7.pdf]

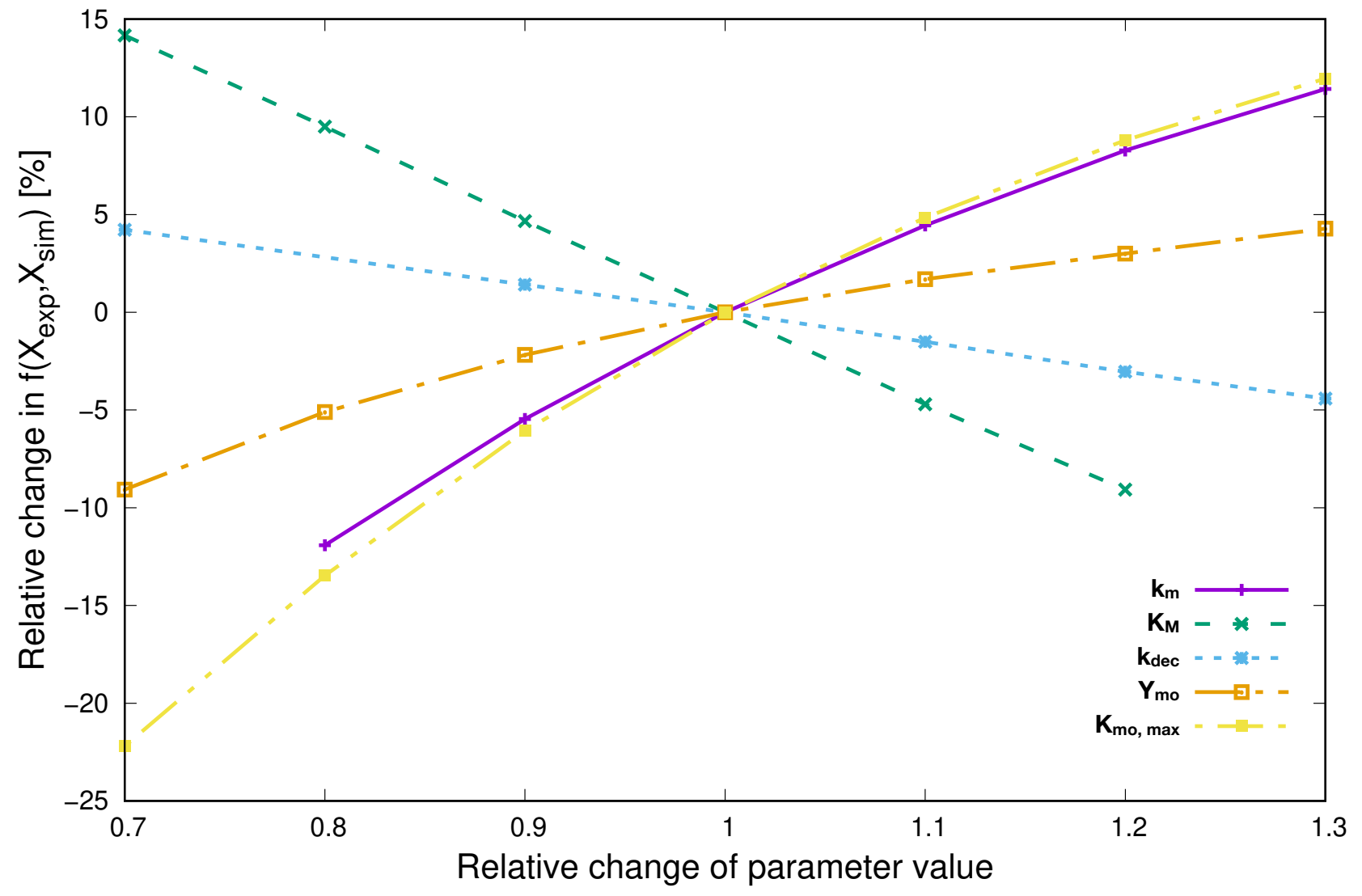

Relative change in f(X_exp,X_sim) [%]
Relative change of parameter value
15
10
5
0
−5
−10
−15
−20
−25
0.7
0.8
0.9
1
1.1
1.2
1.3
k_m
K_M
k_dec
Y_mo
K_mo, max
