# Supplementary material for: Modeling process instability in anaerobic digestion by using a thermodynamically corrected model
Source: Front Bioeng Biotechnol. 2026 Aug 4;14:1880446. doi: 10.3389/fbioe.2026.1880446 (PMC13481626; doi:10.3389/fbioe.2026.1880446)
Supplement: Supplementary file 13 [file DataSheet8.pdf]

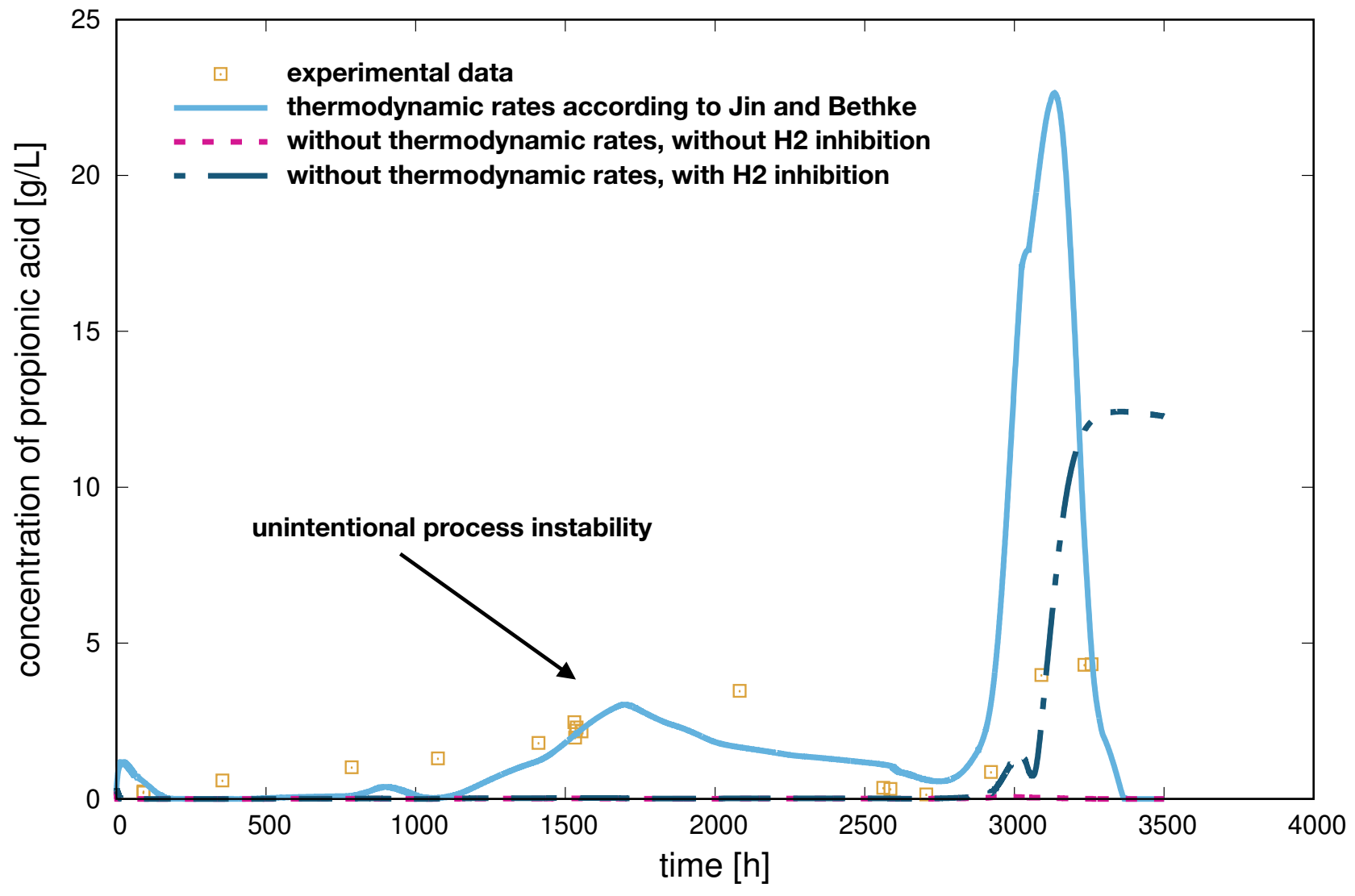

experimental data
thermodynamic rates according to Jin and Bethke
without thermodynamic rates, without H2 inhibition
without thermodynamic rates, with H2 inhibition
unintentional process instability
concentration of propionic acid [g/L]
time [h]
0
5
10
15
20
25
0
500
1000
1500
2000
2500
3000
3500
4000
