# Supplementary material for: Modeling process instability in anaerobic digestion by using a thermodynamically corrected model
Source: Front Bioeng Biotechnol. 2026 Aug 4;14:1880446. doi: 10.3389/fbioe.2026.1880446 (PMC13481626; doi:10.3389/fbioe.2026.1880446)
Supplement: Supplementary file 14 [file DataSheet9.pdf]

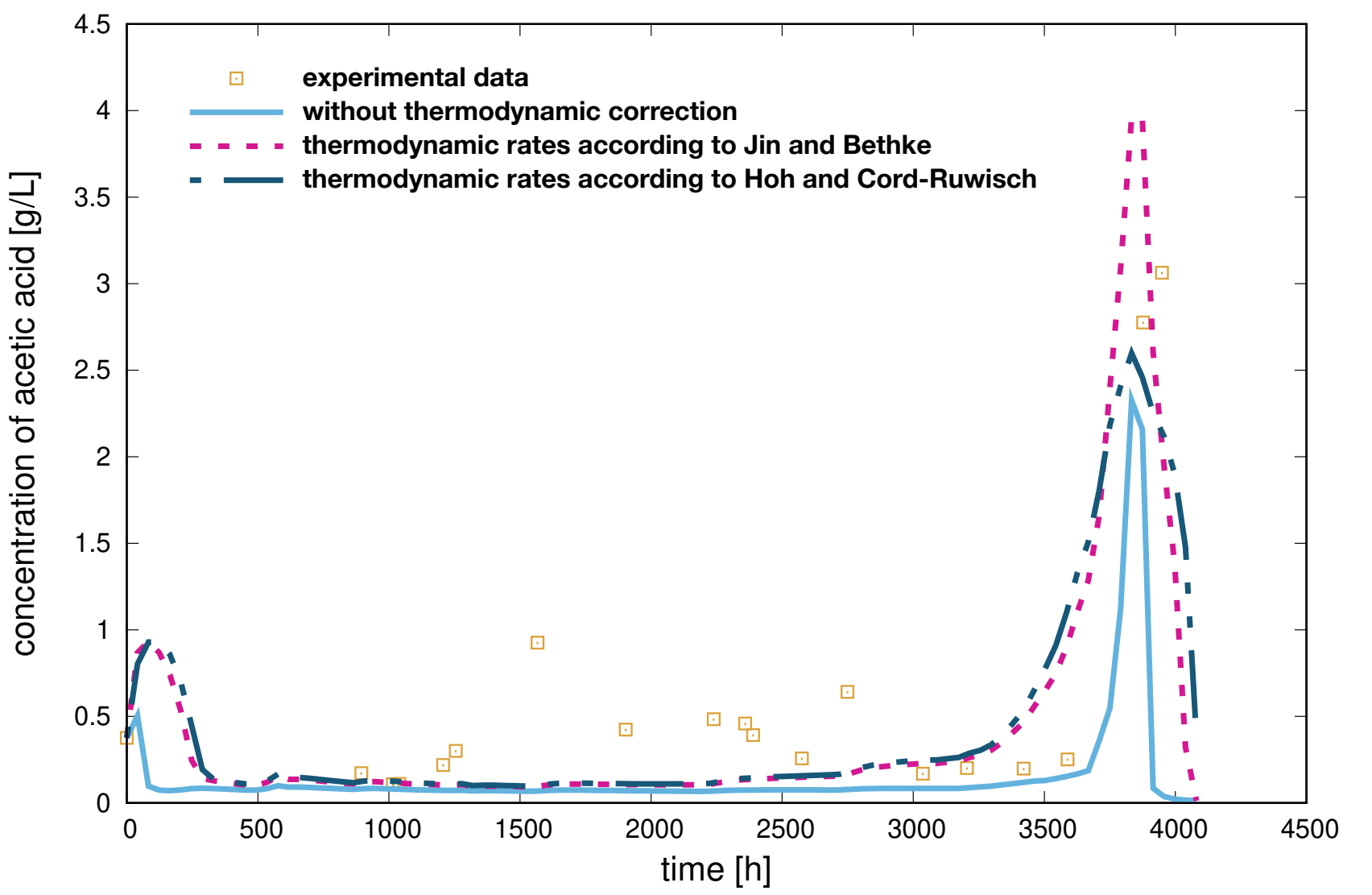

experimental data
without thermodynamic correction
thermodynamic rates according to Jin and Bethke
thermodynamic rates according to Hoh and Cord-Ruwisch
concentration of acetic acid [g/L]
time [h]
0
0.5
1
1.5
2
2.5
3
3.5
4
4.5
0
500
1000
1500
2000
2500
3000
3500
4000
4500
